# Protein Composition of Infectious Spores Reveals Novel Sexual Development and Germination Factors in *Cryptococcus*

**DOI:** 10.1371/journal.pgen.1005490

**Published:** 2015-08-27

**Authors:** Mingwei Huang, Alexander S. Hebert, Joshua J. Coon, Christina M. Hull

**Affiliations:** 1 Department of Biomolecular Chemistry, School of Medicine and Public Health, University of Wisconsin-Madison, Madison, Wisconsin, United States of America; 2 Department of Chemistry, University of Wisconsin-Madison, Madison, Wisconsin, United States of America; 3 Genome Center of Wisconsin, University of Wisconsin-Madison, Madison, Wisconsin, United States of America; 4 Department of Medical Microbiology & Immunology, School of Medicine and Public Health, University of Wisconsin-Madison, Madison, Wisconsin, United States of America; University College Dublin, IRELAND

## Abstract

Spores are an essential cell type required for long-term survival across diverse organisms in the tree of life and are a hallmark of fungal reproduction, persistence, and dispersal. Among human fungal pathogens, spores are presumed infectious particles, but relatively little is known about this robust cell type. Here we used the meningitis-causing fungus *Cryptococcus neoformans* to determine the roles of spore-resident proteins in spore biology. Using highly sensitive nanoscale liquid chromatography/mass spectrometry, we compared the proteomes of spores and vegetative cells (yeast) and identified eighteen proteins specifically enriched in spores. The genes encoding these proteins were deleted, and the resulting strains were evaluated for discernable phenotypes. We hypothesized that spore-enriched proteins would be preferentially involved in spore-specific processes such as dormancy, stress resistance, and germination. Surprisingly, however, the majority of the mutants harbored defects in sexual development, the process by which spores are formed. One mutant in the cohort was defective in the spore-specific process of germination, showing a delay specifically in the initiation of vegetative growth. Thus, by using this in-depth proteomics approach as a screening tool for cell type-specific proteins and combining it with molecular genetics, we successfully identified the first germination factor in *C*. *neoformans*. We also identified numerous proteins with previously unknown functions in both sexual development and spore composition. Our findings provide the first insights into the basic protein components of infectious spores and reveal unexpected molecular connections between infectious particle production and spore composition in a pathogenic eukaryote.

## Author Summary

Spores are a critical cell type that allow long-term survival of diverse organisms from bacteria to fungi to plants. Among fungi, spores are often formed when growth conditions are poor; spores can then disperse to more favorable environments and reinitiate growth. Spores of some environmental fungi can cause fatal disease in humans. Here we used the meningitis-causing yeast *Cryptococcus neoformans* to determine the roles of spore-enriched proteins in spore biology. Using a combined proteomics-genetics approach, we identified eighteen spore-enriched proteins, knocked out the genes encoding each of them, and assessed the resulting strains for phenotypes in a broad array of assays. We predicted that mutant strains would be likely to show defects in spore-specific processes, but instead, we discovered that the majority harbored defects in sexual development, the process by which spores are formed. Only one mutant exhibited a defect in a spore-specific process (germination). Our data reveal that many spore-represented proteins are associated with pre-spore developmental processes, rather than intrinsic spore-specific properties or processes. These findings indicate a previously unknown molecular connection between the developmental process that results in spore biogenesis and the composition of infectious spores.

## Introduction

The formation of survival structures in response to adverse conditions is an essential tool used by diverse organisms across biology to propagate life on earth. Spores are a particularly successful cell type used by many microorganisms, including bacteria, fungi, and protozoa to survive unsuitable growth conditions and/or to disperse to new environments [1]. Among eukaryotes, some of the most environmentally resistant spores are those of fungi, and much of our current understanding of spores comes from studies in model fungi such as *Saccharomyces cerevisiae* and *Aspergillus nidulans* [2]. There are two general categories of fungal spores—sexual and asexual, and both forms occur across diverse fungal species via myriad developmental strategies. For example, in the budding yeast *S*. *cerevisiae* sexual spores are formed when yeast diploids are subject to nitrogen starvation and a nonfermentable carbon source, resulting in four haploid ascospores; *S*. *cerevisiae* does not produce asexual spores [3,4]. In contrast, the filamentous fungus *Aspergillus nidulans* produces both asexual and sexual spores via the development of multicellular fruiting structures with thousands of spores per structure [5]. In all cases, spores are adapted for general survivability.

As a consequence, the basic characteristics of fungal spores are constant: First, mature spores are relatively metabolically quiescent, allowing them to remain dormant for long periods of time under sub-optimal growth conditions (e.g. in the absence of nutrients) [2]. Second, spores are resistant to environmental stresses, such as high temperatures, desiccation, and UV radiation, thus facilitating long-term survival and/or dispersal across great distances around the globe [1]. Third, upon encountering growth-promoting environments, spores can rapidly escape quiescence and germinate to resume vegetative growth [5,6]. As such, spores have evolved to facilitate survival of fungal species in diverse environments, contributing to nearly ubiquitous representation of fungi across all ecosystems on earth.

Spore-producing fungi commonly generate spores with thick, protective coats and robust stress resistance, due to the accumulation of protective solutes (e.g. mannitol and trehalose) and the production of heat shock proteins and other factors that are important for both spore stability and dormancy [2]. Spores respond to different environmental signals to initiate germination, depending on their adapted niches. For example, spores of *S*. *cerevisiae* germinate readily in response to the presence of a fermentable carbon source [6], whereas spores of *Talaromyces macrosporus* require nutrients and a rigorous external trigger of very high temperature or pressure [7,8]. These triggers generally result in responses such as water uptake, cell wall remodeling, and activation of nutrient metabolism and protein synthesis, leading to active fungal growth [5].

The transition from dormant particle to actively growing cell is particularly important because fungal survival cannot occur in the absence of the ability to germinate when (and only when) appropriate for vegetative growth. Environmental fungi are well adapted to their niches, and interestingly, these adaptations have led to a handful of fungi with the ability to cause life-threatening diseases in humans. *Histoplasma capsulatum*, *Blastomyces dermatitidis*, *Aspergillus fumigatus*, *Coccidioides immitis*, *Sporothrix schenkii*, *Penicillium marneffei*, and *Cryptococcus neoformans* all represent environmental fungi that can cause disease in humans, and the most common route of infection is through the inhalation of cells from environmental sources [9]. Spores (sexual or asexual, depending on the fungus) are the most likely infectious particles for all of these pathogens; however, very little is known about their basic spore biology, making the development of disease prevention and treatment strategies challenging.

Among human fungal pathogens, the most common cause of fatal disease (and a well-developed model for study) is *Cryptococcus neoformans*, a primarily opportunistic pathogenic yeast, which causes meningoencephalitis [10]. People with AIDS are particularly susceptible, and there are an estimated one million cases and 600,000 deaths annually worldwide from cryptococcosis [11]. *C*. *neoformans* is ubiquitous in the environment, and inhalation of aerosolized spores and/or yeast is the most common route of infection of humans [12,13]. Under laboratory conditions, spores are produced through sexual development between haploid yeast of opposite mating types (**a** and α) or by α fruiting. In response to specific environmental conditions, cells form filaments and fruiting bodies (basidia) from which haploid, recombinant spores bud in chains [14,15].

Spores of *C*. *neoformans* exhibit the fundamental properties of most fungal spores such as stability in the absence of nutrients and resistance to a variety of environmental stresses, such as high temperature, desiccation, and oxidative stress [16]. These spores have also been shown to germinate efficiently and synchronously in response to nutrients, and they germinate and cause disease in a mouse inhalation model of infection [17,18]. These findings indicate that *C*. *neoformans* spores harbor intrinsic properties that facilitate survival in the environment, maintain spore viability and stability, and initiate germination in response to external signals, including those of a mammalian host.

One approach to understanding how spore-specific properties and behaviors are conferred in *C*. *neoformans* and lead to disease is to identify molecular components that contribute to spore biology. We hypothesized that proteins specific to spores would be more likely to contribute to spore-specific properties than proteins in other cell types (such as yeast). The yeast of *C*. *neoformans* are the vegetative growth form and are physically distinct from spores. They also do not exhibit the same environmental resistance, dormancy properties, or germination processes as spores. To test our hypothesis directly, we carried out a proteomic analysis of *C*. *neoformans* spores and yeast to identify proteins found preferentially in spores that could contribute to fundamental spore behaviors. Here, we present the overlapping and distinct proteomes of both spores and yeast. Using these datasets, we identified spore-enriched proteins, knocked out a cohort of genes encoding eighteen proteins identified only in spores, and assessed the resulting mutants for a wide array of phenotypes. While we anticipated that these spore-enriched proteins would act in spore-specific processes (e.g. stability, resistance, and germination), we discovered instead that the majority of mutants showed defects in early sexual development and spore formation. Our data indicate that many spore-represented proteins are associated with pre-spore and spore formation events during sexual development rather than in conferring intrinsic spore-specific properties, suggesting that spore-resident proteins function in both multi-cellular development and subsequent progeny survival.

## Results

### Protein identification using pure populations of spores and yeast

**Fig 1:**
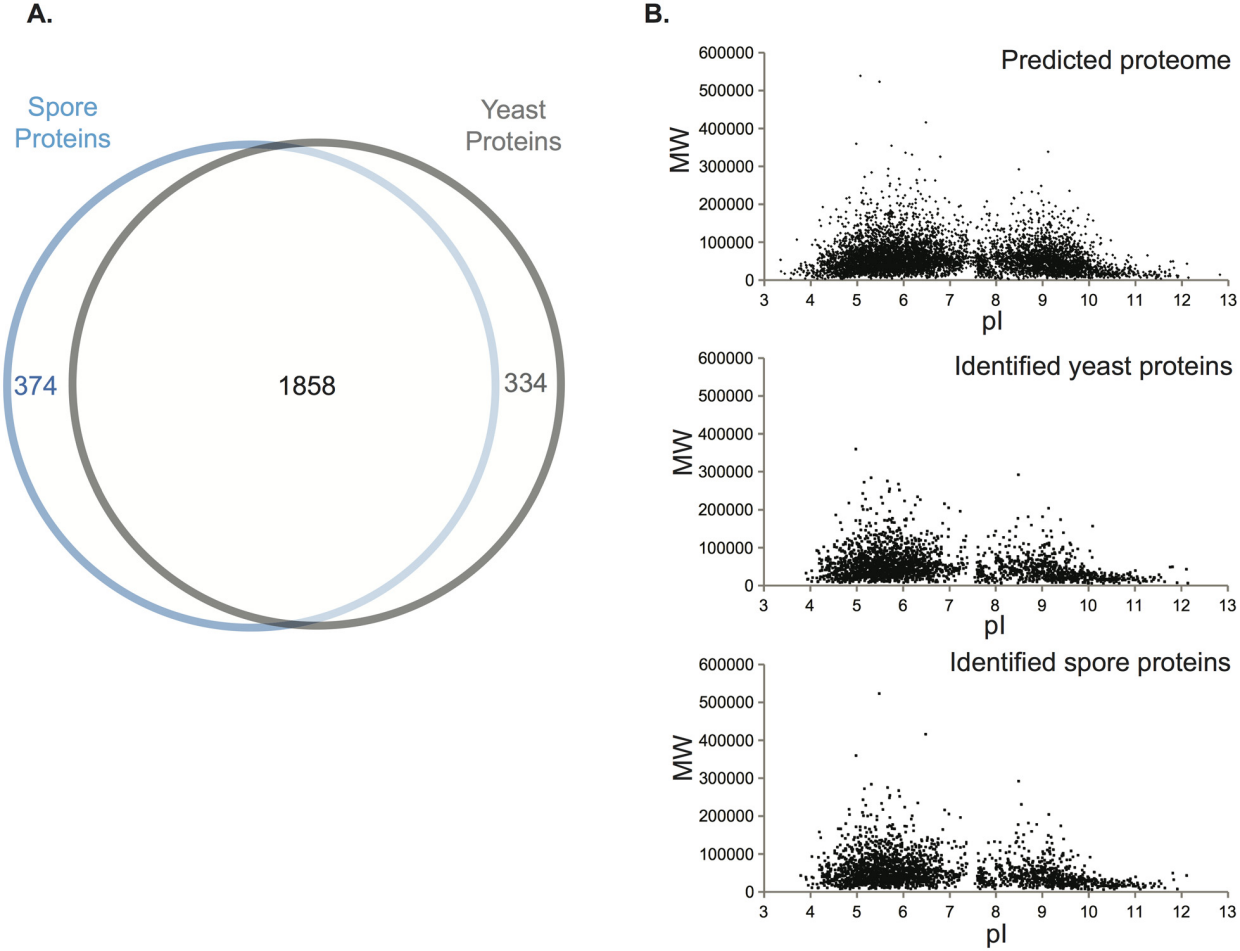
Assessment of the proteomic data. (A) Venn diagram of spore proteins and yeast proteins identified. 2232 and 2192 proteins were identified from spores and yeast, respectively, with a majority of 1858 existing in both cell types. (B) Virtual two-dimensional gel diagrams of the predicted *C*. *neoformans* proteome (upper panel) and identified proteins in either yeast (middle panel) or spores (lower panel). Each dot represents a protein, with the x-axis showing isoelectric point (pI) and the y-axis as molecular weight (MW, Dalton).

To determine differences in protein composition between spores and yeast, we carried out a proteomic comparison using gel fractionation/nanoscale liquid chromatography coupled to tandem mass spectrometry (nanoLC-MS/MS). Proteins were extracted from spores and yeast independently in triplicate. All extracts were subjected to one dimensional sodium dodecyl sulfate polyacrylamide gel electrophoresis (1D SDS-PAGE) analysis (S1A and S1B Fig), trypsin digestion, and high performance liquid chromatographic fractionation/automated tandem mass spectrometry (LC-MS/MS). The resulting mass spectra were evaluated against *C*. *neoformans* proteome databases for peptide and protein identification at a 1% false discovery rate. More than 2000 proteins out of the ~6500 predicted proteins of *C*. *neoformans* were identified for both spores and yeast, and there was ~80% overlap in protein composition between the two cell types (Table 1 and Fig 1A and S1 Table).

**Table 1:** Number of proteins identified in spores and yeast.

Experiment	Spore	Yeast	Spore-enriched^a^	Yeast-enriched^b^
Replicate 1	1798	1868	345	415
Replicate 2	1277	1265	239	227
Replicate 3	1639	1563	343	267
Total non-redundant^c^	2232	2192	374	334
Overlap among replicates	976	995	18	16

^a^. Proteins identified only in spores but not in yeast

^b^. Proteins identified only in yeast but not in spores

^c^. Proteins identified in at least one replicate of spores or yeast

To assess the overall quality of our data, we evaluated the distribution of the molecular weights and isoelectric points, the predicted intracellular locations, and the general molecular functions of all the proteins encoded by the genome and compared those to the proteins in our datasets. The distribution patterns for the proteins identified by mass spectrometry were similar to the predicted proteome overall (Fig 1B, S1C and S1D Fig). Transmembrane proteins were the only major exceptions: 39.4% of the *C*. *neoformans* genome is predicted to encode transmembrane domains, but only 21.7% of the proteins in our dataset were predicted to harbor transmembrane domains. This amount of bias was consistent with previous proteomic studies using MS, indicating that no additional bias was introduced during protein extraction or recovery in our experiment [19]. The proteins detected in our experiment also included all of the proteins from previously published proteomic studies of *C*. *neoformans* secreted and cell wall-bound proteins as well as immunodominant proteins (S2 Table) [20,21]. Total numbers of proteins identified were also similar to other MS analyses of *C*. *neoformans* (Table 1) [22]. Overall, these findings indicate that the quality of the yeast and spore datasets is high and very likely to accurately reflect the proteomes of each cell type.

### *C*. *neoformans* spores and yeast harbor both overlapping and distinct proteomes

**Fig 2:**
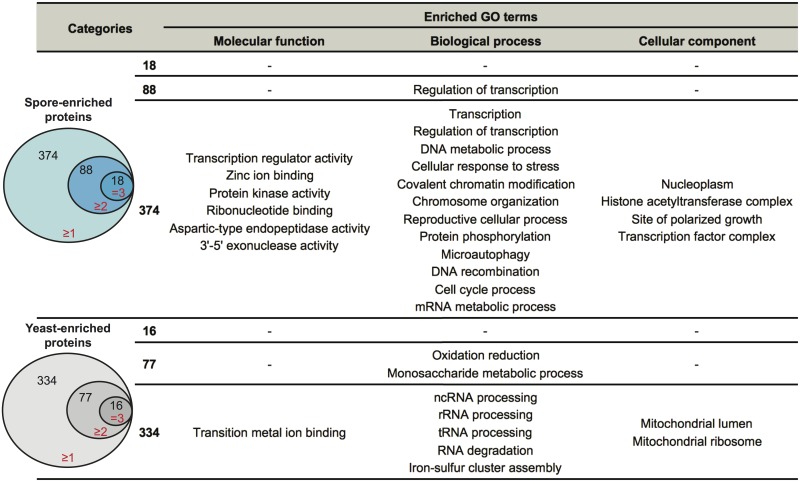
Enriched biological processes in spore-enriched or yeast-enriched proteins. Spore-enriched proteins were defined as those identified only in spores but not in yeast in our datasets. There were 374 spore-enriched proteins (blue circles) identified in at least one replicate, 88 in at least two replicates, and 18 in all three replicates. Yeast-enriched proteins (grey circles) are defined as those identified only in yeast but not in spores in our dataset. There are 334 yeast- enriched proteins identified in at least one replicate, 77 in at least two replicates, and 16 in all three replicates. To the right are enriched specific GO terms in each group. Functional annotation clustering analysis was performed using DAVID. Only significantly enriched (p<0.05) GO terms are listed.

To identify proteins likely to confer cell type-specific properties, we evaluated the spore and yeast proteomes for differences using two approaches. First, we assessed proteins in a binary manner, determining only their presence or absence in all datasets. We defined proteins that were detected in at least one spore sample, but never in a yeast sample as "spore-enriched" and vice versa. Using this method, we identified 374 spore-enriched proteins. Within this group of 374 proteins, 88 and 18 proteins were detected in two or three spore replicates, respectively, but not in any yeast replicates (Table 1 and Fig 2 and S3 Table). Conversely, we detected 334 "yeast-enriched" proteins in at least one replicate, 77 in two, and 16 in all three yeast replicates, but never in any spore samples (Table 1 and Fig 2 and S3 Table).

To evaluate the likely functional properties of yeast- and spore-enriched proteins, we carried out functional annotation clustering analysis, using the Database for Annotation, Visualization and Integrated Discovery (DAVID) [23,24]. Enrichment analysis showed that when compared to all 2560 proteins that were identified at least once in yeast or spores, the 374 spore-enriched proteins were significantly overrepresented (p<0.05) in a variety of specific GO clusters, such as regulation of transcription, DNA metabolic processes, chromosome organization, and mRNA metabolic processes (Fig 2). In contrast, analysis of the 334 yeast-enriched proteins revealed overrepresentation in iron-sulfur cluster assembly, mitochondria function, and RNA processing and degradation (Fig 2). These patterns of representation are consistent with the concept of the spore as a cell type poised to respond to changing environmental conditions and of the yeast as an actively growing vegetative cell type.

In the second analysis, we used spectral counting to estimate relative abundances of each protein in spores and yeast (rather than a binary readout of presence or absence in the sample). Spectral counting is particularly useful for estimating large differences in protein composition between samples and can provide more accurate estimates than stable isotope labeling methods [25,26]. Specifically, normalized peptide spectral match (PSM) values were used to calculate a spore-overrepresentation ratio (r) for each protein (r = spore PSM value divided by yeast PSM value). We designated 156 spore-overrepresented proteins (r>4) and 317 yeast-overrepresented proteins (r<0.25). Spore-overrepresented proteins show GO term enrichment similar to that of spore-enriched proteins, but enriched terms also included fungal cell wall, external encapsulating structure, and Golgi apparatus. Yeast-overrepresented proteins also showed GO term enrichment similar to yeast-enriched proteins but enriched terms also included specific biosynthetic and metabolic processes (Table 2). Thus, the addition of yeast- and spore-overrepresented protein categories resulted in the identification of additional differences in GO categories in biological processes and cellular components between yeast and spores. These data further distinguish differences between the yeast and spore cell types at a molecular level.

**Table 2:** Spore-overrepresented and yeast- overrepresented proteins.

			Enriched specific GO terms		
Categories	Spore enrichment ratio	Number of proteins	Molecular function	Biological process	Cellular component
**Spore-overrepresented proteins**	> 4	156	ion binding, peptidase activity	chromosome organization, chromatin modification, regulation of transcription, cell cycle process	fungal type cell wall, external encapsulating structure, Golgi apparatus part
**Yeast-overrepresented proteins**	< 0.25	317	iron ion binding	Organic acid biosynthetic process, hexose metabolic process, carbohydrate biosynthetic process, sulfur metabolic process, lipid biosynthetic process	Mitochondrion

Functional annotation clustering analysis was performed using the Database for Annotation, Visualization and Integrated Discovery (DAVID). The underlined GO terms expand those identified in spore-enriched proteins (Fig 2).

### Spore-enriched proteins fall into diverse functional categories

Given the unique role of spores in microbial survival, we focused on proteins likely to be important for spore-specific functions. We hypothesized that spore-enriched proteins would be more likely than other proteins to be involved in spore-specific processes such as dormancy, stress resistance, and germination. Thus, we focused on the 18 proteins that showed a spore-enriched MS identification pattern in all three spore proteome replicates. To verify that the spore-enriched pattern represented in the mass spectrometry analysis was reflected in the levels of protein in vivo, we created two strains harboring fusions between spore-enriched proteins and a fluorescent protein, mCherry. In both cases, mCherry fluorescence was visible only in spores and not in yeast, consistent with the proteomic data (S2 Fig). We assigned the genes encoding the 18 spore-enriched proteins gene names based on similarity to known genes in *S*. *cerevisiae* when possible and placed them in five groups based on predicted functions (Table 3). Group 1: Replication and Chromosome Biology, 2: Transcription and Splicing, 3: Cellular Transport, 4: Carbohydrate Metabolism, and 5: Proteins of Unknown Function. Seven genes encoding proteins with no similarity to previously named proteins were named Identified Spore Protein (*ISP1-7*).

**Table 3:** Eighteen genes encoding spore-enriched proteins.

Gene	JEC21 ID	Predicted functions/domains	Deletion phenotype(s)
**Group 1: Replication and Chromosome Biology**			
*TOP1*	CNI03280	topoisomerase I	sporulation defect
*IRR1*	CNA07890	nuclear cohesin complex component	inviable
**Group 2: Transcription and Splicing**			
*RSC9*	CNB00580	chromatin remodeling complex component	cell fusion defect
*DST1*	CNF01160	general transcription elongation factor TFIIS	sporulation defect
*PRP31*	CNB05520	U4/U6-U5 snRNP complex component	inviable
*PRP11*	CND02290	SF3a splicing factor complex component	inviable
**Group 3: Cellular Transport**			
*BCH1*	CNG02530	specialized cargo export from Golgi	filamentation defect
*SFH5*	CNE04320	non-classical phosphatidylinositol transfer protein	no phenotype
*DDI1*	CNC00460	vSNARE binding protein	sporulation defect
*EMC3*	CNF02470	protein folding in the ER	decreased spore yield
**Group 4: Carbohydrate Metabolism**			
*GRE202*	CNG01830	D-lactaldehyde dehydrogenase	decreased spore yield
*ISP1* ^*a*^	CNB02490	conserved in fungi/short chain dehydrogenase	filamentation defect
*ISP3*	CND04560	conserved in fungi/mannose-6-phosphate isomerase	no phenotype
*ISP4*	CNK01510	conserved in fungi/glycosyl hydrolase	no phenotype
**Group 5: Proteins of Unknown Function**			
*ISP2*	CNE01730	*Cryptococcus*-specific/no conserved domains	increased sporulation; slow germination
*ISP5*	CNB04980	conserved in fungi/ferritin-like superfamily domain	no phenotype
*ISP6*	CNA04360	*Cryptococcus*-specific/transmembrane domain	no phenotype
*ISP7*	CND00650	*Cryptococcus*-specific/no conserved domains	no phenotype

^a^. Genes encoding proteins with no obvious homologs were named *ISP* for Identified Spore Protein.

To test our hypothesis, we deleted the entire open reading frames of genes encoding these 18 spore-enriched proteins in haploid yeast of both mating types, and the resulting mutants were evaluated for discernable phenotypes. Among the eighteen genes, 3 appeared to be essential because we were repeatedly unable to recover transformants that did not harbor a wild type copy of the targeted gene. The homologs of these genes in *S*. *cerevisiae*, *IRR1*, *PRP31*, and *PRP11*, are all known to be essential for viability [27–29]. Among the other fifteen genes that were deleted, all of the resulting mutants were viable, including *top1Δ*. This was surprising because *TOP1* has been shown previously to be essential for viability in *C*. *neoformans* strain H99 (serotype A) [30]. *TOP1* was readily deleted in the JEC20 and JEC21 strains (serotype D) used here. Because *TOP1* is not essential for survival in many fungi [31,32], we surmised that unknown differences between serotype backgrounds may account for this difference in phenotype within *C*. *neoformans*. None of the other genes had been characterized previously in *C*. *neoformans*.

**Fig 3:**
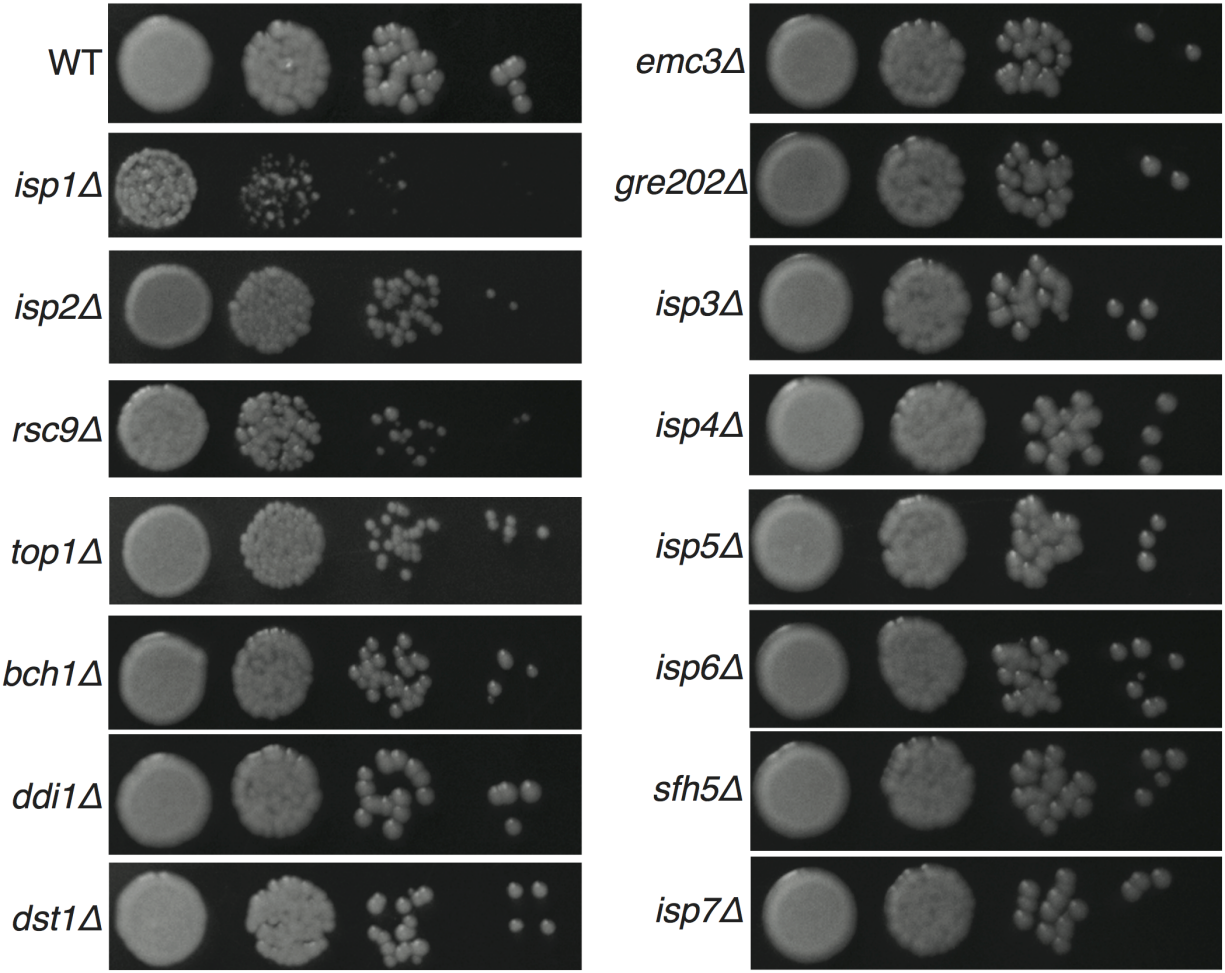
Yeast growth on YPD solid medium at 30°C. Yeast of the same starting concentration were spotted at 10-fold serial dilutions and grown for 3 days at 30°C. Multiple independent mutant strains were tested and representative growth for each mutant is shown.

To assess general yeast growth of the 15 viable gene deletion strains, we evaluated growth of multiple independent knockout strains for each in both mating types at 30°C under nutrient-rich growth conditions (YPD agar). Eleven mutants grew in a manner indistinguishable from that of wild type strains, three (*isp2Δ*, *rsc9Δ* and *top1Δ*) showed slightly slower growth, and only one (*isp1Δ*) showed a substantial growth defect (Fig 3). Growth at 25 and 37°C generally followed the same pattern (S3 Fig). For all of the mutant strains, growth rates relative to wild type strains in YPD liquid culture at 30°C were also similar to growth on solid agar (S4 Fig). Given the generally robust growth of the deletion strains on solid agar at multiple temperatures, we were confident that the strains could be assessed accurately in further phenotypic analyses and reveal phenotypes independent of basic vegetative growth.

### Many spore-enriched proteins are required for normal sexual development

**Fig 4:**
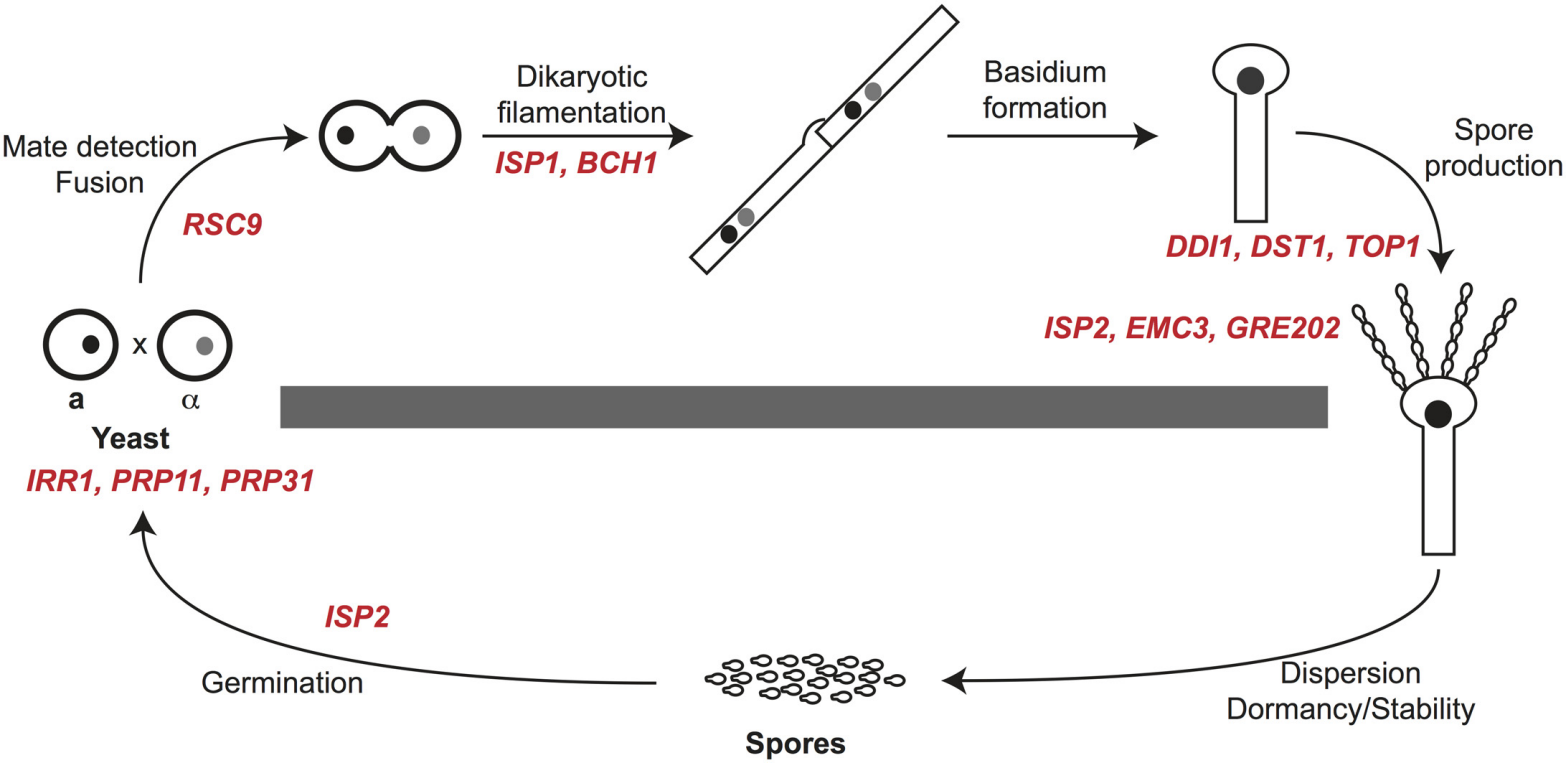
Summary of spore-enriched protein mutant phenotypes. *C*. *neoformans* spores are produced through sexual development (above the gray bar). Sexual development occurs between haploid yeast of opposite mating types (**a** and α) and includes mate detection and cell fusion, dikaryotic filamentation, basidium formation and production of spores in chains. Spores can disperse and germinate into yeast (below the gray bar) to complete the life cycle. Gene names in red text are placed at the process in which deletion mutants show phenotypes. Six genes, *ISP3-7* and *SFH5*, did not result in detectable phenotypes in any assays.

To assess the ability of mutants to produce spores, we assessed the behavior of viable deletion mutants in crosses (**a** mutant × α mutant). In *C*. *neoformans* mating between **a** and α cells initiates a form of sexual development that is robust under laboratory conditions (V8 agar at 25°C for 5–7 days) and results in filamentation, fruiting body (basidium) development, and spore formation. We evaluated crosses between **a** and α strains of each mutant for defects in development delineated in details below. To summarize, we discovered that eight of the fifteen mutants displayed defects in sexual development. One mutant showed severe defects in cell fusion (*rsc9Δ*), two were defective in filamentation (*isp1Δ* and *bch1Δ*), three showed severe defects in spore formation (*ddi1Δ*, *dst1Δ*, and *top1Δ*), and two yielded fewer spores than wild type strains (*emc3Δ* and *gre2Δ*). One mutant (*isp2Δ*) showed more robust filamentation and increased spore biogenesis (Fig 4 and Table 3). The remaining mutants (*sfh5Δ*, *isp3Δ*, *isp4Δ*, *isp5Δ*, *isp6Δ*, and *isp7Δ*) did not show any discernible phenotypes in sexual development.

**Fig 5:**
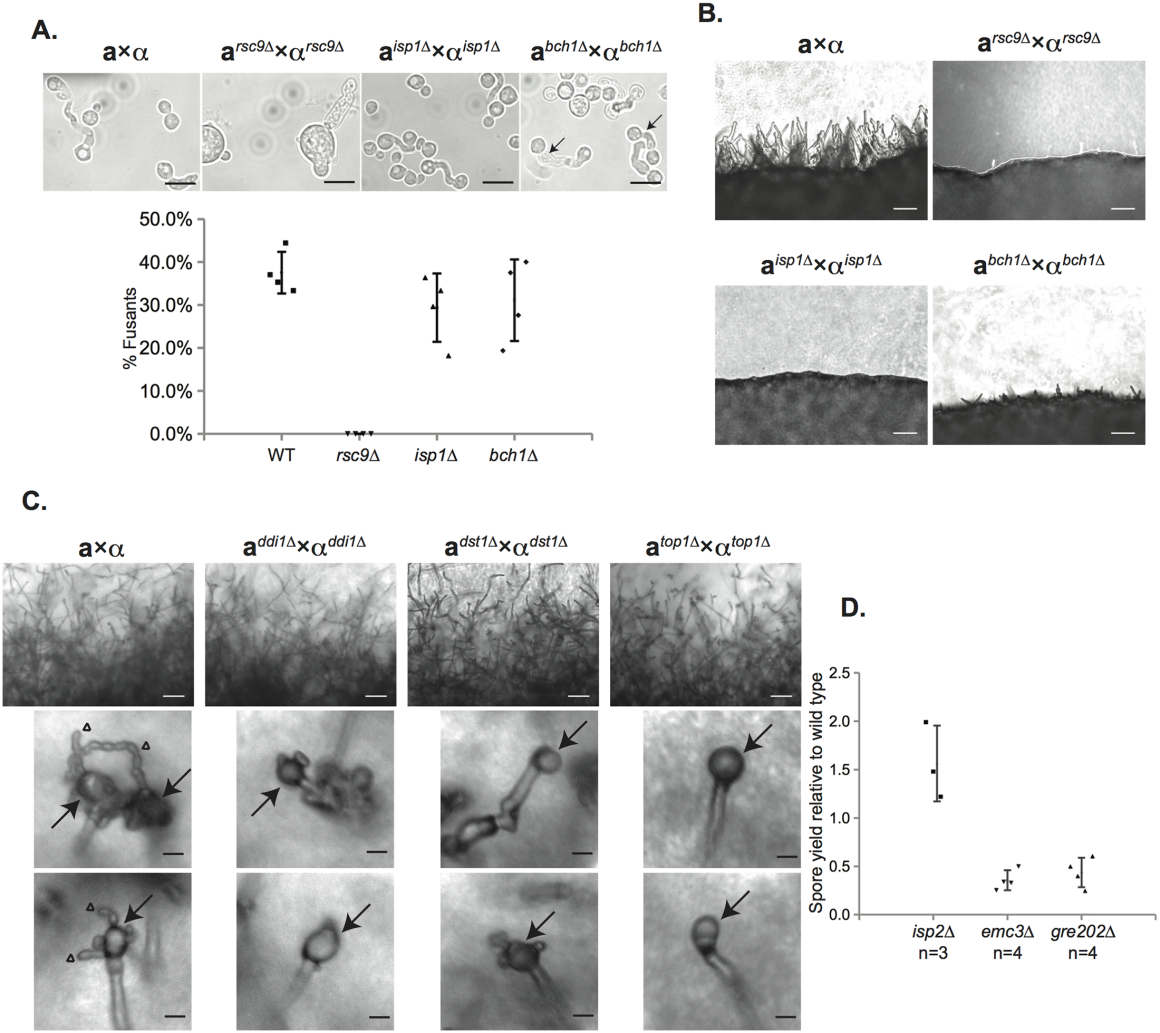
Characterization of the sexual development of deletion mutants for spore-enriched proteins. (A) *rsc9Δ* strains show defects in mating, while *isp1Δ* and *bch1Δ* strains do not (top panels). Strains were mixed, incubated on V8 plates for 24h at 25°C, scraped up and visualized. Fusants (indicated by arrows) and yeast were counted. The number of fusants as a portion of total cell number is represented graphically for each strain (bottom panel). Data represent four individual experiments and are shown as mean ± standard deviation (SD). Scale bars, 10μm (100x magnification). (B) *rsc9Δ*, *isp1Δ*, and *bch1Δ* crosses showed much less robust filamentation 24h after the start of sexual development. Panels show the periphery of a spot of a cross on V8 plate for 24h at 25°C. Scale bars, 50μm (200x magnification). (C) *ddi1Δ*, *dst1Δ*, and *top1Δ* strains showed defects in spore formation. Both wild type and mutant crosses showed robust filamentation after 5 days on V8 plate (upper panels), but only wild type produced chains of spores (lower panels; arrows indicate basidia and triangles indicate spore chains). Mutants produced basidia without spore chains. Scale bars, 50μm (200x magnification) for upper panels and 10μm for lower panels (400x magnification). Spore isolations using density gradient centrifugation yielded 1%±1%, 2%±1%, and 0%±0% spores relative to wild type crosses from *ddi1Δ*, *dst1Δ*, and *top1Δ* crosses, respectively. (D) Quantified spore yield from density gradient purifications of mutant strains relative to wild type strains. *emc3Δ* and *gre202Δ* crosses yielded reproducible decreases in spore yield (approx. 2–4 fold) relative to wild type strains, whereas *isp2Δ* crosses produced more spores (approx. 1.5-fold higher) relative to wild type strains. Data represent number (n) of independent experiments and are shown as mean ± SD.

For each mutant sexual development was evaluated microscopically to assess the formation of developmental structures. Crosses between mutant strains were initiated and evaluated after 24 hours for the presence of classic fusant structures and filaments. One mutant, *rsc9Δ*, showed a severe defect in the ability to undergo cell fusion and no fusants were observed (Fig 5A). This inability to initiate sexual development resulted in an absence of subsequent filamentation (Fig 5B). Two strains (*isp1Δ* and *bch1Δ*) formed fusants at frequencies indistinguishable from wild type strains (Fig 5A), but both showed severe defects in filament formation along with *rsc9Δ* (Fig 5B and S5 Fig).

The remaining six mutant strains (*top1Δ*, *dst1Δ*, *ddi1Δ*, *emc3Δ*, *gre202Δ*, and *isp2Δ*) all formed robust filaments and were assessed microscopically for basidium and spore formation. All of the strains formed basidia in a manner indistinguishable from wild type. However, spore formation was severely affected in three mutants; crosses between *ddi1Δ*, *dst1Δ*, and *top1Δ* strains formed basidia, but only few or no visible spores, consistent with significant reductions in spore yields when purified using density gradient centrifugation (Fig 5C) [16]. Higher resolution microscopy of the filaments of these three mutants revealed apparent defects in nuclear migration (S6 Fig) In contrast, *emc3Δ*, *gre202Δ*, and *isp2Δ* crosses all formed robust spores. To quantitate the relative number of spores from each, we carried out spore isolations via gradient centrifugation and quantitated the number of spores formed per cross. We discovered that two strains, *emc3Δ* and *gre202Δ*, consistently yielded 2–4 fold fewer spores than wild type crosses, whereas the third strain, *isp2Δ*, yielded ~50% more spores than wild type (Fig 5D).

### Mutant spores are not less stable or less resistant to stress-inducing conditions than wild type spores

To evaluate the roles of identified spore proteins in conferring spore-specific properties, we purified spores from crosses and subjected them to a series of assessments of their basic morphological properties, ability to survive stressful environmental conditions, and ability to remain dormant under non-germinating conditions. Spores were recovered from crosses of all of the mutants that produced sufficient numbers of spores for analysis (*i*.*e*. all mutant strains except *rsc9Δ*, *bch1Δ*, *isp1Δ*, *top1Δ*, *dst1Δ*, and *ddi1Δ*). In all cases spores from the mutant strains were indistinguishable from wild type spores with respect to basic morphology and surface composition as determined by both light and fluorescence microscopy (S7A Fig). Spores were also evaluated for their ability to survive high temperature (50°C), oxidative stress (20 mM H_2_O_2_), and longer-term viability (growth after 8 weeks at 4°C in PBS). Spores from the mutant strains exhibited survival properties identical to those of wild type spores under these conditions (S7B Fig). Finally, we assessed spores for overall stability (ability to remain dormant) by incubating them in PBS at 30°C for 120 hours and recording any changes in morphology and germination ability over that time. Again, no differences were detected between mutant and wild type spores (S7B Fig).

### *isp2Δ* spores show a delay in germination

To identify roles that spore-enriched proteins might play in the spore-specific process of germination, mutant spores were grown under a variety of conditions and evaluated for the ability to germinate and form a colony. Spores were grown on YPD at 25°C and 30°C, under nutrient limiting conditions (YP, SD, filament agar), and on YPD in the presence of cell wall stressors (Congo Red, caffeine, or SDS) or an osmotic stressor (NaCl). All of the mutant spores grew into colonies under all conditions tested at a frequency indistinguishable from wild type spores (S7C Fig).

**Fig 6:**
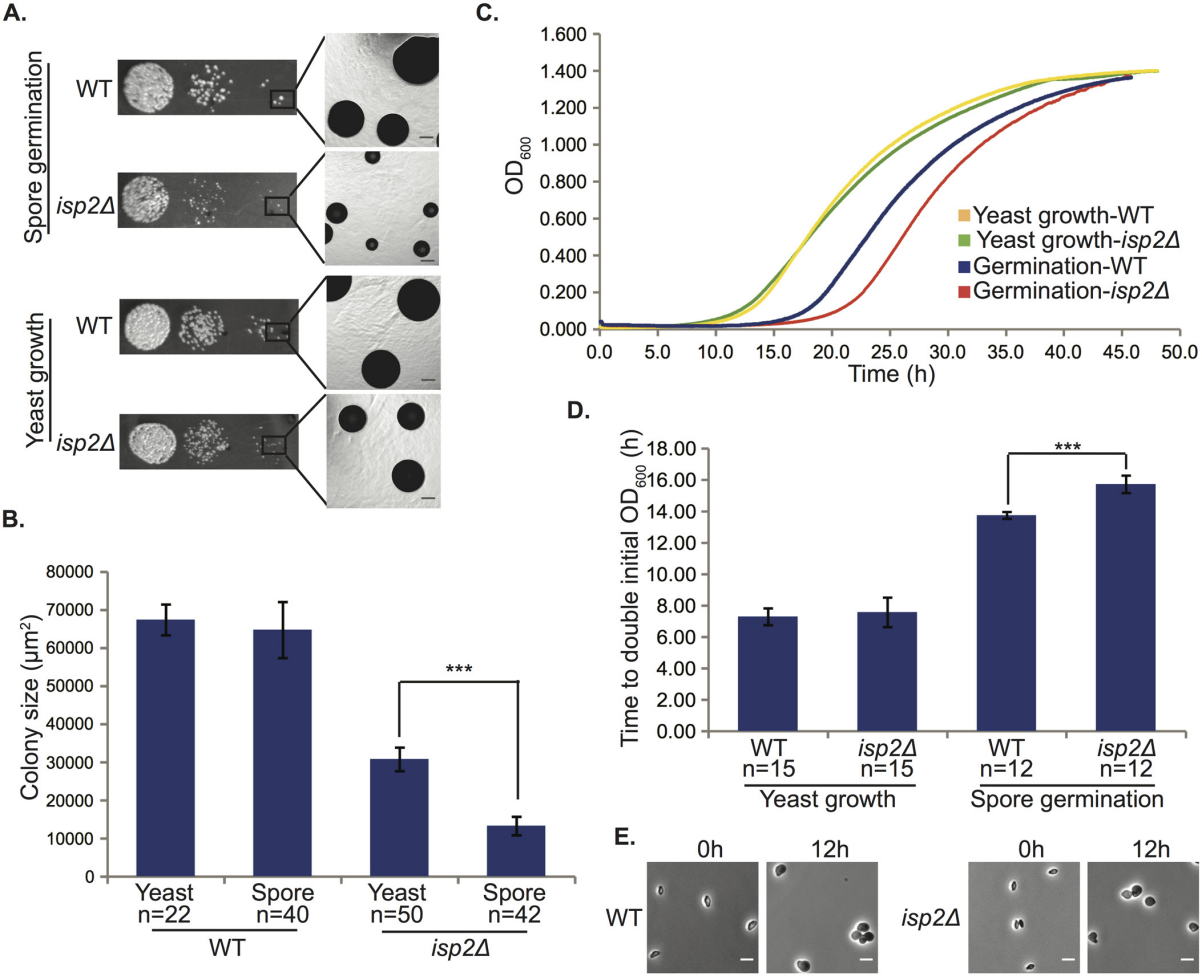
*isp2Δ* spores show a delay in germination. (A) *isp2Δ* spores have a germination defect on solid YPD medium. Colonies of wild type and *isp2Δ* strains after growth at room temperature for 63h (germination) and 51h (vegetative growth). Scale bars, 100μm (5x magnification). (B) Quantification of colony sizes using ImageJ. The average colony size of *isp2Δ* spores was only 20.5% of wild type spores, whereas the average colony size of *isp2Δ* yeast was 45.7% of wild type yeast. The difference in size between colonies from yeast growth and spore germination for *isp2Δ* strains was significant (p = 7.1x10^-48^) but not for the wild type strain (p = 7.8x10^-1^). Data represent number (n) of independent experiments and are shown as a mean ± SD. An unpaired two-sided Student's t-test was used to assess significance. (C) Germination delay for *isp2Δ* spores in liquid YPD media. Optical density at a wavelength of 600nm (OD_600_) was measured every 3min over 50h. The y-axis shows OD_600_ and the x-axis shows time in hours (h). Plots are representative of three independent experiments. (D) Average time taken to double initial OD_600_. Quantified doubling times were nearly identical for wild type and *isp2Δ* yeast (p = 0.38); however, *isp2Δ* spores took significantly longer than wild type to double the population (p = 1.2×10^−10^). Data represent number (n) of independent experiments and are shown as mean ± SD. An unpaired two-sided Student's t-test was used to assess significance. (E) Morphological changes during germination of *isp2Δ* and wild type spores. Spores were exposed to YPD liquid media to trigger germination at room temperature and photographed at 0h and 12h. Scale bars, 5μm (1000× magnification).

However, germinated spores of one mutant (*isp2Δ*) consistently produced smaller colonies relative to wild type spores (Fig 6A, top). Because yeast of the *isp2Δ* strain grew at a slower rate than wild type yeast on agar plates (Figs 3 and 6A, bottom), we surmised that the differences observed between *isp2Δ* and wild type spores during germination could be a consequence of differences in yeast growth rates subsequent to germination. In wild type strains, colonies from germinating spores are smaller than those from yeast due to the delay caused by germination itself (12h). To account for the time required for germination and identify differences in colony size specific to germination in the mutant strain, we adjusted the growth times for yeast and spores (51h vs. 63h) to normalize the yeast and spore colony sizes for the wild type strain (Fig 6A, WT yeast growth vs. WT spore germination). We then quantitated the difference in colony size between yeast and spores of the *isp2Δ* strain and identified a significant difference in size between yeast- and spore-derived colonies from the *isp2Δ* strain under these conditions (~50% decrease in colony size). These data suggested that the *isp2Δ* strain harbored an additional growth phenotype associated specifically with germination (Fig 6B).

To confirm this observation we also carried out germination assays in liquid culture. We had observed previously that the modest slow-growth phenotype of *isp2Δ* yeast was limited to growth on agar plates and did not occur in liquid culture (S4 Fig). Thus, we carried out quantitative germination assays in liquid culture to evaluate the *isp2Δ* spores under conditions that would suppress any differences in yeast growth. Spores and yeast from stationary growth phase cultures were seeded into YPD liquid medium and grown at room temperature for 50h, and OD_600_ was measured every 3min. We observed a clear delay in the growth of *isp2Δ* spores relative to wild type spores, whereas there was no significant difference in growth between *isp2Δ* and wild type yeast (Fig 6C and 6D). Quantified doubling times were nearly identical for wild type and *isp2Δ* yeast (7.3±0.5h vs. 7.6±0.9h); however, it took 13.8±0.2h for wild type spore germination cultures to double the initial cell concentration and 15.7±0.6h for *isp2Δ* mutants, resulting in an ~2h delay for the mutant in achieving log phase growth, suggesting that *ISP2* plays a role in spore germination. To exclude the possibility that deletion of *ISP2* resulted in changes in expression of nearby genes, leading to the observed phenotype, we assessed levels of gene expression of *ISP2* and its neighbors in both wild-type and mutant strains via qRT-PCR. As expected, levels of *ISP2* transcript were easily detected in the wild type strain and undetectable in the *isp2Δ* strain. In contrast, transcript levels of the genes upstream and downstream of *ISP2* in the *isp2Δ* strain were indistinguishable from the wild type strain, indicating that disruption of *ISP2* did not affect neighboring loci (S8 Fig). From these data we conclude that *ISP2* is responsible for the germination delay and plays a specific role in spore germination.

The apparent difference in germination rate between wild type and *isp2Δ* spores suggested several possibilities: a delay in the initiation of germination, a slower rate of germination (i.e. a decrease in rate of differentiation from a spore into a yeast), or a delay in entering regular vegetative growth. To differentiate among these possibilities, we microscopically evaluated individual spores of both wild type and *isp2Δ* strains every four hours from 0h to 16h after the initiation of germination. We observed that the *isp2Δ* spores were identical to wild type spores in their abilities to initiate, sustain, and complete the process of germination (110 and 122 spores from wild type and *isp2Δ* strains were evaluated, respectively). By 12h the morphological transition from spore to yeast was complete and indistinguishable between wild type and *isp2Δ* spores (Fig 6E). Based on these observations, it appears that the delay leading to smaller colonies during germination occurs after the morphological transition to yeast. These findings indicate that the *isp2Δ* mutant spores were delayed between the end of the morphological transition from spore to yeast and the beginning of active growth. This is consistent with the liquid germination assays in which there was a two-hour delay in *isp2Δ* mutant entry into log phase growth. As such, we can pinpoint a delay in growth of the *isp2Δ* mutant spores at the transition from a fully formed yeast to a vegetatively reproducing yeast, confirming a role for Isp2 in the spore-specific process of re-initiating vegetative growth in response to nutrients during germination.

**Fig 7:**
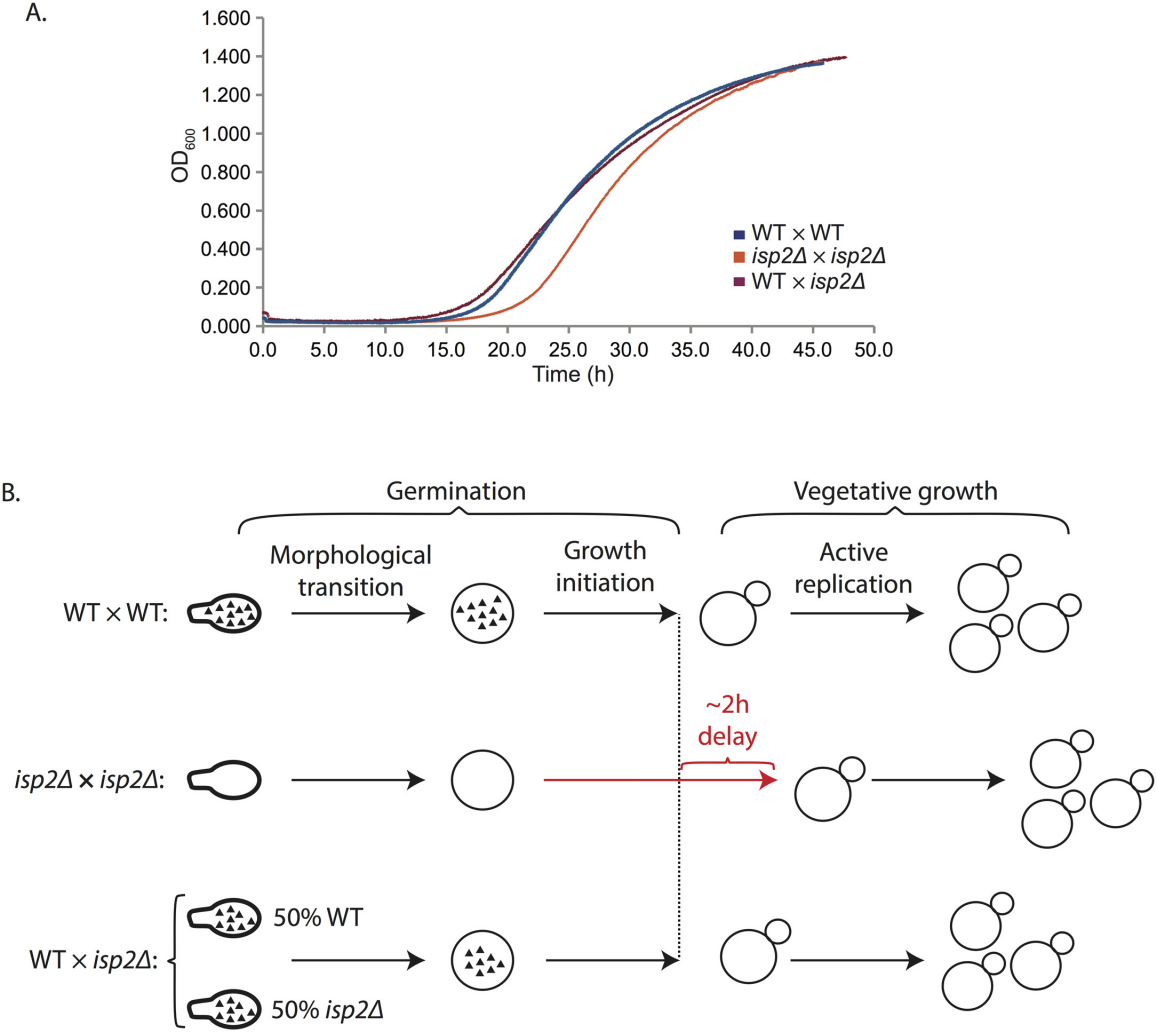
Spores derived from wild type by *isp2Δ* crosses show wild type rates of germination. (A) Spores purified from WT × *isp2Δ* strains were grown in YPD and measured via optical density (OD_600_) every 3min over 50h. The y-axis shows OD_600_ and the x-axis shows time in hours (h). Plots are representative of three independent experiments. (B) Model of Isp2 activity during germination. Germination encompasses two stages: a morphological transition and growth initiation before the active replication of vegetative growth. Isp2 protein (black triangles) is present in mature spores from WT × WT crosses and persists during germination through the morphological transition to contribute to optimal growth initiation. In contrast, there is no Isp2 in spores from *isp2Δ* × *isp2Δ* crosses, and thus, a delay of ~2h during germination occurs, specifically during the growth initiation phase. Notably, spores from WT × *isp2Δ* crosses do not show a delay in germination and therefore contain Isp2 protein similar to wild type spores, regardless of genotype. Spores are shown as ovals with stalks, whereas yeast are shown as spheres. Large and small spheres together represent budding yeast. Isp2 protein is represented by black triangles.

To determine the source of Isp2 activity during germination (i.e. basidium-derived vs. spore-derived), we also carried out liquid germination assays with spores purified from crosses between wild type and *isp2Δ* strains (WT × *isp2Δ*). This population of spores contains a 50:50 ratio of wild type to *isp2Δ* genotypes. We predicted that if the Isp2 protein necessary for wild type germination were produced by spores, then half of the spores from a wild type by *isp2Δ* cross would harbor the germination delay phenotype (causing a shift in the liquid germination assay curve that would fall between those of WT × WT and *isp2Δ* × *isp2Δ* spores). In contrast, if the Isp2 necessary for normal germination were basidium-derived (deposited in spores during spore biogenesis), then the presence of Isp2 in the mutant spores would lead to a germination curve identical to wild type spores. We observed that spores from crosses between wild type and *isp2Δ* strains germinated at a rate indistinguishable from wild type spores, indicating that Isp2 function is not impaired in this mixed-genotype population (Fig 7A). These findings suggest that the Isp2 protein detected in spores is produced in the basidium and deposited into spores during spore formation, resulting in subsequent wild type germination in the absence of the *ISP2* gene (Fig 7B).

## Discussion

In this study we determined the spore and yeast proteomes of *C*. *neoformans* and discovered 18 proteins that were consistently represented in spores and not in yeast. We hypothesized that these proteins would be likely to participate in spore-specific functions such as dormancy, stress resistance, and germination. Surprisingly, however, we discovered that the eight out of nine mutants with discernible phenotypes exhibited defects during sexual development. Five of the mutants displayed defects in spore biogenesis, indicating that the composition of spores reflects (at least in part) the proteins necessary to create a spore. The ninth mutant showed a defect in the critical spore-specific process of germination. Thus, by using an unbiased proteomics approach as a screening tool, we successfully identified proteins whose functions are important throughout sexual development and in spore biology. None of these proteins had been associated previously with sexual development or spore biology, and very few molecular components that function during sexual development are known. Our findings provide the first glimpses into the composition of spores and the interplay between sexual development and the functions of a key resulting infectious cell type.

### Fungal spore proteomes are diverse

Proteomic analyses of spores have been used in an array of fungal species, and studies have identified proteins associated primarily with the biological processes of protein synthesis, protein folding and degradation, and metabolism and energy production [19,33]. In *C*. *neoformans* spores, we detected proteins involved in these same categories, but we found no significant differences in enrichment of these categories between yeast and spores. Instead, our proteomic comparisons between spores and yeast indicated that spore proteins are enriched in distinct processes, including regulation of transcription, DNA metabolic processes, chromosome organization, and mRNA metabolic processes. Comparing spores with vegetative growth forms provides the advantage of being able to ascribe spore-specific features, properties, or functions to any given class of proteins and improve resolution of spore-relevant pathways. For example, analyses of *Aspergillus fumigatus* conidia (asexual spores) and mycelia (vegetative form) revealed that conidia disproportionately harbor proteins associated with reactive oxygen intermediates (ROI) detoxification, pigment (melanin) biosynthesis, and conidial rodlet layer formation [34]. In contrast, in the related species *Aspergillus nidulans*, proteins related to ROI detoxification and some heat shock proteins were more abundant in mycelia than in conidiospores [35]. In *C*. *neoformans*, there were no differences between spores and yeast in any of these categories, suggesting that there are large differences in survival strategies across fungal species. Perhaps the different conditions leading to spore formation contribute to distinct proteomes among species or between asexual and sexual spores. Comprehensive proteomic studies to link the well-characterized morphological changes, underlying molecular events, and transcriptional networks that control sporulation in many fungi will be extremely useful in providing a global understanding of how the spore proteome is synthesized through the corresponding developmental process.

### Spore proteins are required for many stages of sexual development

In *C*. *neoformans* sexual development between cells of opposite mating types involves five distinct morphological events: mate detection, cell fusion, filamentation, basidium formation, and sporulation. We predicted that proteins overrepresented in spores would be likely to participate in subsequent spore processes such as dormancy, stress resistance, and germination. Instead, we discovered that nearly half of the mutants in spore-enriched proteins showed phenotypes in development *prior* to the formation of spores. None has been associated previously with fungal development or spore formation, and these proteins fell into highly diverse biological and functional categories.

For example, we discovered that Rsc9 is required for haploid yeast to fuse with one another to initiate sexual development. In *S*. *cerevisiae* and *Schizosaccharomyces pombe* Rsc9 is one component of the RSC chromatin remodeling complex, and it is required for viability in both [36,37]. In *S*. *cerevisiae RSC9* is involved in genome-wide transcriptional response to a stress-induced signaling cascade [38], and in *S*. *pombe RSC9* is down-regulated when cells are subjected to nitrogen limitation [39]. Although in *C*. *neoformans RSC9* is not required for vegetative viability, the slower growth of *rsc9Δ* mutants indicates its involvement in vegetative growth [40]. One possibility is that the dramatic defect observed during sexual development is due to a role for *RSC9* in a regulatory response to nutritional stresses encountered during development.

Of the two genes we found required for early filamentous growth, *ISP1* and *BCH1*, the latter likely participates in active intracellular trafficking during filamentation. Bch1 is a member of the ancient family of Chs5p-Arf1p-binding Proteins (ChAPs) that are conserved in fungi and required for export of specialized cargo from the Golgi [41]. In *S*. *cerevisiae* one important cargo, chitin synthase III (Chs3), is required for mating projection formation and chitosan production in ascospore walls. More importantly, filamentous fungal growth requires the endocytic system that transports secretive vesicles and early endosomes, through the cytoskeleton in a variety of fungi [42]. Thus, it is possible that Bch1 is required for the transport of specific cargos necessary for filamentation during sexual development of *C*. *neoformans*, leading to abnormal vacuole distribution and/or accumulation and disruptions in nuclear movement in *bch1Δ* strains (S5A Fig). The other filamentation gene, *ISP1*, contains several conserved NAD(P) binding sites and is annotated as a putative short chain dehydrogenase, but no significant homologs have been identified in other organisms. In *S*. *cerevisiae*, mitochondrial/metabolic functions are required for the transition to a filamentous form under certain conditions of nutrient stress [43]. One possibility in *C*. *neoformans* is that Isp1 contributes to overall energy production through a development-specific redox reaction during filamentation that is important to drive filament formation and proper nuclear migration (S5A Fig).

Overall, the discovery of Rsc9, Bch1, and Isp1 as important players in early sexual development was surprising and suggests that proteins with high representation in spores could be "left over" from previous steps in development and passively carried into newly produced spores (but not necessarily function in spore processes). Alternatively, these proteins could serve multiple roles in development and reflect a highly efficient, intercalated process in which proteins serve critical roles at multiple stages. In this case, sexual development proteins could function not only during fusion and filamentation but also later during spore biogenesis and be actively deposited into spores for spore-specific processes. For example, it is known that histone modifications and packaging of chromatin take place during spore formation in other organisms [3]; thus, it is plausible that Rsc9 plays roles in both signaling during mating and chromatin condensation during spore biogenesis in *C*. *neoformans*.

### Spore biogenesis is dependent on spore-enriched proteins

In response to unknown signals, filamentation during sexual development ends, and a basidium forms on the terminal filament. None of the mutants in genes encoding spore-enriched proteins showed phenotypes specific to basidium formation. However, the majority of mutant strains with detectable phenotypes (5 in total) showed defects during spore biogenesis. Three genes *TOP1*, *DST1*, and *DDI1* were essential for spore formation, and mutants formed basidia that were largely devoid of spores and appeared "bald." This phenotype has been observed previously for only two other genes in *C*. *neoformans*, *SPO11* and *UBC5*, which are conserved meiosis genes in fungi [44]. The absence of spores in *spo11Δ* and *ubc5Δ* strains suggests that meiosis and spore formation are tightly coupled (as has been observed in other fungi). This would be consistent with our finding that mutants in *TOP1*, a critical topoisomerase, do not form spores likely as a result of improper decatenation of meiotic products.

Ddi1 in *S*. *cerevisiae* is a DNA damage-inducible SNARE binding protein that has been found to be involved in cell-cycle control and repression of protein secretion in vegetative cells of *S*. *cerevisiae* (but does not play a significant role in sporulation) [45,46]. Ddi1 in *C*. *neoformans* shares all of its conserved domains at high similarity with its *S*. *cerevisiae* homolog, but it is clearly required for spore formation. One possibility is that the formation of hundreds of spores per basidium in *C*. *neoformans* requires Ddi1 partitioning of limited vesicle resources in a manner not required for the four ascospores of *S*. *cerevisiae*.

*DST1* encodes the conserved general transcription elongation factor TFIIS, and is required for the balanced expression of genes encoding ribosomal components under transcriptional stress [47]. Deletion of *DST1* in *S*. *cerevisiae* leads to a slight defect in sporulation [46]; however, in *C*. *neoformans DST1* is indispensable for efficient spore production, perhaps reflecting higher demands for ribosomal components needed by the basidium to produce spore components.

Deletion of genes encoding two spore-enriched proteins, Emc3 and Gre202, led to no visible defect in sexual development or spore formation. However, gradient centrifugation purification of spores from *emc3Δ* and *gre202Δ* crosses consistently and repeatedly revealed decreases in spore yields of 2–4 fold (Fig 5D). In other fungi Emc3 is a member of a conserved endoplasmic reticulum (ER) membrane protein complex, which contributes to efficient protein folding in ER and whose deletion induces the unfolded protein response (UPR) [48]. Gre202 is an NADPH-dependent methylglyoxal reductase, potentially capable of reducing a very wide spectrum of compounds [49]. It remains to be determined how these proteins contribute to spore yields and other spore-related processes.

### Three proteins essential for viability were overrepresented in spores

We designated the proteins found only in spores and not in yeast in our datasets as "spore-enriched" because they could be either truly specific to spores or simply highly overrepresented in spores relative to yeast. This possibility was borne out by the discoveries of Irr1, Prp11, and Prp31 in the spore proteome data (and not in the yeast data). All three of these proteins are essential for viability in other organisms, and our failed attempts to knock out the genes encoding them in *C*. *neoformans* were consistent with these proteins playing essential roles in *C*. *neoformans* as well. Why these proteins are overrepresented in spores relative to yeast is not entirely clear; however, in *S*. *cerevisiae*, Irr1 is a subunit of the essential cohesin complex, which is needed for sister chromatid cohesion during mitosis and meiosis [50], and Prp11 and Prp31 are necessary for pre-mRNA processing, which can contribute to meiosis-specific splicing of certain messages for the control of gene expression during sporulation [51,52]. It is plausible that given the demands for meiosis and repeated mitosis in the *C*. *neoformans* basidium prior to spore biogenesis, these proteins are at high levels in the basidium and are carried into developing spores. Alternatively, Prp11 and Prp31 may be deposited in spores to facilitate splicing of transcripts necessary for germination.

### Germination of fungal spores

One of the 18 mutant strains displayed a spore-specific phenotype; the *isp2Δ* shows a delay in spore germination at the step in which vegetative growth is initiated. The protein sequence of Isp2 is 374 amino acids, and shows no similarity to any other known or predicted proteins or protein domains in any organism (with the exception of another weakly related protein in *C*. *neoformans* (CNM00430)). As such, no clear structures or functions can be gleaned from sequence information; however, the Protein Homology/analogY Recognition Engine V 2.0 (Phyre2) algorithm [53] suggests the possibility of a trihelix DNA binding. Therefore, Isp2 might function as a transcription factor during germination, but this remains to be determined.

Discerning the function of Isp2 in the process of germination will be important in future studies in part because of the paucity of information available regarding fungal germination. Germination of spores is a critical process required for propagation of fungal species, and among fungal pathogens, germination is essential for causing disease. However, there are long-standing questions about how fungal spores germinate. Relative to other cellular processes, we know very little about the molecular mechanisms governing spore germination, and it is not yet clear whether germination is a specific process with specific machinery or if it is a somewhat modified exit from a form of stationary phase growth into log phase growth.

Few spore proteins have been identified to be involved specifically in spore germination in the fungal kingdom. In *S*. *cerevisiae*, members of the ras/mitogen-activated protein kinase pathway and the transcription factor Ume6 have been found to be important for spore germination, but they also play important roles during vegetative growth and meiosis during sporulation [6,54]. In addition, large-scale genetic screens in *S*. *cerevisiae* for mutants with defects in spore germination have not identified germination-specific components [46,55].

In contrast, our data for Isp2 suggest very strongly that in *C*. *neoformans* germination is distinct from vegetative growth and likely requires at least some germination-specific machinery. The activity of Isp2 also points to a specific germination program because Isp2 does not function in a spore-autonomous manner. Rather, akin to the "maternally deposited" transcripts and proteins of metazoans during embryogenesis, Isp2 appears to be packaged into spores during spore biogenesis. It is this "maternal" protein that governs an efficient transition to vegetative growth during late germination; spores that do not harbor an intact *ISP2* gene behave like wild type spores during germination as long as they were derived from crosses between wild type and mutant strains (WT × *isp2Δ*). As such, Isp2 protein and/or transcript must be produced prior to spore formation and packaged into spores. Based on these findings, we propose that Isp2 protein is produced in basidia prior to and/or concurrent with the biogenesis of spores and is deposited specifically into the spores. This is consistent with the overrepresentation of Isp2 protein in spores relative to yeast in our proteomic data. We posit that Isp2 then contributes to efficient spore germination at a specific, late stage of differentiation—after the morphological transition of the small, ovoid spore into a larger, round yeast and before active replication during vegetative growth (growth initiation phase) (Fig 7B). This is consistent with our direct observations of changes in spore morphology and quantitative assessments of spore germination rates and yeast growth.

With the discovery of Isp2, we anticipate that additional germination-specific machinery exists and can be identified using similar proteomic approaches. High sensitivity mass spectrometry (nanoLC-MS/MS) in combination with molecular genetics and quantitative phenotype assays is a powerful tool for assessing protein functions in rare cell types. Ultimately, stage-specific proteomics during germination will enable a high-resolution view of the germination process. The identification and characterization of a germination-specific, developmentally-deposited, spore-resident protein is an unprecedented finding in fungal spores with the potential to provide opportunities for novel pathway discovery. Isp2 and other germination-specific proteins could be promising targets for the development of inhibitors to be used in the prevention of fungal growth and spore-mediated disease.

## Materials and Methods

### Strain manipulations and media

All strains used were of the serotype D background (*Cryptococcus neoformans* var. *neoformans* strains JEC20 and JEC21), and their genotypes are listed in S5 Table [56,57]. All were handled using standard techniques and media as described previously [58,59].

### Protein extraction and preparation for mass spectrometry

Spores were purified from crosses after 6 days on V8 agar using density gradient purification as described previously [16]. Yeast were grown on V8 agar plates for 6 days prior to harvest. Spores or yeast were suspended in lysis buffer (50mM Tris-HCl pH 7.5, 1% SDS, 10mM EDTA) and sonicated 5 times for 12 seconds each at a power output of 2 and 100% duty cycle on ice using Branson Sonifier 250 (Emerson Industrial Automation, USA). Resulting lysates were extracted with 1:1 phenol-chloroform, precipitated with ethanol, and washed with ethanol and acetone. The pellet was air-dried and dissolved in SDS-containing sample buffer [60]. Proteins were separated in a 12% PAGE Bis-Tris gel and visualized using Coomassie Brilliant Blue-staining [61]. Proteins were recovered and prepared for MS as follows: Each protein-containing PAGE gel lane was excised cut into 5 bands and minced. Gel samples were washed with 100mM NH_4_HCO_3_, incubated at ambient temperature, and then dried. The samples were reduced by resuspension in 100mM NH_4_HCO_3_ with 10mM dithiothreitol and incubation at 56°C for 1h. Samples were then treated with 100mM NH_4_HCO_3_ with 55mM iodoacetamide, incubated for 45 minutes in the dark at ambient temperature, washed twice with 100mM NH_4_HCO_3,_ incubated in acetonitrile for 5 minutes, and dried. Samples were digested with 12.5ng/μL trypsin in 100mM NH_4_HCO_3_ for 30min. Peptides were extracted by overnight incubation in 100mM NH_4_HCO_3_, followed by washing with 50mM NH_4_HCO_3_, 50% acetonitrile and 5% formic acid, and 100% acetonitrile. Extracted peptides were dried and resuspended in 15μL 0.2% formic acid.

### LC-MS/MS

Peptides were loaded onto a 75μm inner diameter column packed with 5μm Magic C18 particles (Michrom) and eluted with increasing acetonitrile over 60min. Eluted peptides were ionized by electrospray and analyzed by an LTQ Orbitrap Velos, Velos pro, or an LTQ mass spectrometer. For the MS1 survey scan, 1×10^6^ ions were analyzed by the Orbitrap or 4×10^4^ ions were analyzed by the ion trap. From this scan the 10 most intense features were selected for MS/MS analysis with a 30-60s dynamic exclusion. Fragmentation data was produced by either HCD with analysis in the Orbitrap with 2×10^5^ ion target value, or with CAD with analysis in the ion-trap with 1×10^4^ target value.

### Data analyses

Peptide analysis was performed using the COMPASS software suite. Spectra were converted into text files and searched against the *Cryptococcus neoformans* proteomic database from NCBI, using the OMSSA search algorithm. Precursor average mass was searched with a tolerance set to 3.5Da, monoisotopic fragment ions were searched with tolerance set to 0.5Da or 0.01Da for ion-trap and orbitrap spectra, respectively. Peptides were filtered to 1% FDR based on E-value alone or E-value and ppm mass error for Ion-trap and Orbitrap data, respectively. Peptides were grouped into proteins and filtered to 1% FDR using established rules. The mass spectrometry data from this publication have been submitted to the Chorus project database (https://chorusproject.org/pages) and assigned the project ID 751.

Molecular weights and isoelectric points of proteins were predicted using Compute pI/Mw Tool from the ExPASy server [62]. Transmembrane helices in proteins were predicted using TMHMM version 2.0 [63]. Cellular localizations were predicted using WoLF PSORT [64] and molecular functions by Blast2GO [65]. Functional annotation clustering analyses were performed using the Database for Annotation, Visualization and Integrated Discovery (DAVID) [23,24] and all proteins that were identified at least once in yeast or spores (Table 1 and S1 Table) were used as the background pool for comparison.

Peptide spectral match (PSM) numbers associated with protein identification were used to estimate relative protein abundance. Data were normalized to the global average number of peptides detected per protein per experiment excluding the top and bottom fifth percentile data [66]. PSMs in each experiment were scaled to produce a global average across all experiments. To estimate the relative abundance of each spore protein compared to yeast, the PSM numbers for each protein from each replicate were summed and a ratio (r) of spore PSM to yeast PSM for each protein was determined.

### Generation of deletion strains

Deletion constructs were created using fusion PCR [67] and the primers listed in S5 Table. For each gene the 5'- region was amplified with primers P1 and P2, the 3'-region was amplified with P5 and P6, and the cassette of selection marker was amplified with P3 and P4. PCR fusion using P1 and P6 was used to create the final full-length deletion cassette. The *NAT* ^*R*^ and *NEO* ^*R*^ deletion cassettes were transformed into JEC20 and JEC21 by biolistic transformation, grown on rich medium containing 1M sorbitol, and selected on medium containing 200μg/mL G418 or 200μg/mL nourseothricin [68]. The *URA5* deletion cassettes were transformed into JEC34 and JEC43 also by biolistic transformation, grown on minimum medium containing 1M sorbitol for selection. Resulting colonies were screened using PCR for correct insertion of the knockout construct (P7 and P8) and absence of target ORF (P9 and P10), and further assessed via Southern blotting for single integration of the construct to identify multiple independent knockouts in both mating types [61]. At least three independent **a** and α strains were recovered for each deleted gene. All yeast growth phenotype tests were carried out with at least four independent strains per gene. All sexual development and spore phenotype tests were carried out in at least three pairs of crosses between independent **a** and α strains per gene. All phenotypes were observed in all independent deletion strains tested for each gene of interest.

### Generation of mCherry strains

Recombinant constructs were created using fusion PCR [67] and primers were listed in S5 Table. For each gene, the ORF region immediately upstream of the stop codon was amplified with primers P1 and P2, the downstream region was amplified with P5 and P6, and the cassette of mCherry sequence and selection marker was amplified with P3 and P4. PCR fusion using P1 and P6 was used to create the final full-length transformation cassette. The *mCherry-URA5* replacement cassettes were transformed into JEC34 and JEC43 also by biolistic transformation, grown on minimum medium containing 1M sorbitol for selection. Resulting colonies were screened using PCR for correct insertion of the construct and further assessed via Southern blotting for single integration of the construct to identify multiple independent knockouts in both mating types [68]. At least three independent **a** and α strains were recovered for each gene. Spores were purified from at least three pairs of crosses between independent **a** and α strains per gene. Consistent expressions of recombinant mCherry proteins were observed in all independent strains tested for each gene of interest.

### Growth assays

Deletion strains were grown to stationary phase in liquid YPD culture overnight at 30°C with shaking and then used as seed cultures to assess their growth phenotypes in fresh liquid YPD culture during log phase or on solid YPD plate as described previously [69]. Briefly, the seed cultures were diluted to an OD_600_ of 0.05 in fresh liquid YPD medium and their growth at 30°C with shaking was monitored by taking samples for absorbance measurement at a wavelength of 600nm every two hours in a time course of around twenty-four hours. The growth curves were then plotted and doubling times were calculated based on their growth rates during log phase. The same diluted cultures were also spotted on YPD plates at 10-fold serial dilutions and grown for 3–4 days at room temperature, 30°C, or 37°C before being visually examined and photographed.

### Sexual development assessment

#### Filamentation assays

Mixed haploid yeast of opposite mating types were spotted onto V8 solid medium at a ratio of 1:1 and a total OD_600_ of 1.0. Plates were then incubated at room temperature in the dark and microscopically evaluated over a 7-day period. Light microscopy was carried out on a Zeiss Axioskop 2 fluorescent microscope. Photographs were taken with an Axiocam MRM REV3 digital camera. For fluorescent microscopy, 15μL of 1×PBS with 0.4mg/mL Calcofluor white MR2 (Sigma) or Sytox Green (Life Technologies) to stain cell wall or nuclei was first spotted onto glass slides, then cells were scraped from the plate and suspended in those spots of solution before microscopic examination.

#### Fusion assays

Crosses were scraped off V8 plates after 24h incubation in the dark at room temperature, suspended in PBS, and examined microscopically. Four different fields were randomly chosen for counting fusants and total number of cells to generate fusant:total ratios.

#### Spore yield

Crosses containing equal numbers of cells were spotted in 25μL per spot and 40 spots per plate onto five V8 plates. Spores were purified from 7-day crosses using density centrifugation and then counted using a hemacytometer (Fisher Scientific). Mutant and wild type spores were isolated using the same conditions and the same number of plates. All spore purifications used multiple independent pairs of crosses to determine the final relative yields compared to wild type.

### Spore phenotype assessments

Spores from each mutant were stained with 0.05mM calcofluor white MR2 (Sigma) or 1:50 diluted Concanavalin A (ConA, Vector labs), visualized microscopically, and photographed. Spore germination was assessed on rich solid YPD medium by spotting 4μl 10-fold serial dilutions from a starting concentration of 10^3^/μl and incubating for 3–4 days at room temperature, 30°C, or 37°C before being photographed using a SCION CFW-1309M Grayscale Digital Camera. Germination was also tested under a variety of stressful conditions at 30°C, including nutrient-limiting media (YP no dextrose, Synthetic medium no dextrose, and filament agar) and YPD medium with cell wall stressors (1mg/ml Congo Red, 0.1mg/ml caffeine, and 0.005% SDS) or osmotic stressors (1M NaCl). To test their heat stress resistance, spores were incubated at 50°C for 5 or 10 minutes before spotting on YPD plate and germinating at 30°C as described above. Oxidative stress resistance was tested by incubating in 20mM hydrogen peroxide for 5 minutes prior to washing and germination [16].

### Reverse-transcriptase PCR

Wild type, *isp2Δ*, and complemented crosses were initiated on V8 plates as described earlier and incubated for 48h or 72h before RNA extraction. Then RNA was extracted using a hot phenol method [61] and cleaned up using RNeasy Mini Kit (Qiagen). First-strand cDNA synthesis was carried out using Superscript III reverse transcriptase with 5μg total RNA and oligo(dT)_12-18_ according to manufacture’s manual (Invitrogen). Quantitative realtime PCR (qRT-PCR) was performed using the Bio-Rad CFX96 real-time system with a C1000 thermal cycler (Bio-Rad). Each PCR reaction was in triplicate and used 5ul diluted cDNAs, SYBR Green (Sigma), and oligo pairs listed in S5 Table. The expression level of each gene is normalized to the internal reference gene *GPD1* and relative to wild type. All values were generated by Bio-Rad CFX manager software v. 2.0.

### Quantitative germination assays

#### Solid agar

Wild type and *isp2Δ* mutant spores or yeast were grown on YPD 63h or 51h, respectively at room temperature so that resulting colonies were of the same size. Photographs were taken with a SCION CFW-1309M Grayscale Digital Camera and an Axiocam MRM REV3 digital camera. ImageJ was used for image processing and quantification of colony size (algorithm Huang for thresholding, watershed if needed, 0.85 circularity, 1000 minimal size). Twenty-two wild type and 50 *isp2Δ* colonies from yeast and 40 wild type and 42 *isp2Δ* colonies from spore germination were assessed for their size and statistical significance was calculate using unpaired two-ended Student's t-test. Liquid: 100μl of spore suspensions in YPD liquid media were grown in a 96-well plate (Corning Life Sciences) in triplicate for each strain and each initial concentration (starting OD_600_ of 0.012, 0.018, 0.024, 0.030, and 0.036). OD_600_ was measured every 3 minutes for 48 hours at 25°C using a Synergy 2 multi-mode microplate reader controlled by Gen5 software (BioTek Instruments). Spores from two pairs of *isp2Δ* crosses and one pair of wild type crosses were tested. Parallel yeast cultures were inoculated similarly with an initial OD_600_ of 0.010. Doubling times were determined manually. Direct observations of germination and outgrowth were carried out in YPD liquid media at 25°C. Aliquots of germination suspension were visually assessed at 0h, 12h, 14h, and 16h using a Zeiss Axioskop 2 microscope.

## Supporting Information

S1 Fig Proteomic identification and analysis of both spores and yeast in *C*. *neoformans*. (A) Schematic workflow of protein exaction. A density-gradient centrifugation method was used to purify large quantities of spores from crosses between **a** and α cells. 1×10^9^ spores were sonicated to release proteins, which were subsequently precipitated by organic solvent. The resulting pellet was boiled in SDS-containing buffer to dissolve protein for SDS-PAGE analysis. (B) Protein extracts from yeast and spores were analyzed by SDS-PAGE. Coomassie Brilliant Blue-staining showed that sufficient amount of proteins (about 100μg estimated by staining) were obtained. (C) Distribution of subcellular localization for all the proteins encoded by the genome, identified in spores or yeast. Their localizations were predicted using WoLF PSORT. Plasma membrane proteins are under-represented and cytoplasmic proteins are correspondingly over-represented in our dataset of identified spore or yeast proteins. (D) Distribution of general molecular function for all the proteins encoded by the genome, identified in spores or yeast. Molecular functions were predicted and analyzed using Blast2GO.
(TIFF)

S2 Fig Fluorescently tagged, spore-enriched proteins Isp2 and Isp7 in spores, yeast, and basidia. mCherry was fused to the C-termini of Isp1 and Isp7 and expressed under their endogenous promoters. (A) Spores and yeast or (B) basidia harboring each recombinant expression construct were visualized using fluorescence microscopy and photographed. Left panels are merged images of light field and red fluorescence channel. Right panels are images from the red fluorescence channel only. Isp1-mCherry, showed visible fluorescence in spores, but no levels of fluorescence over background in other cell types, including basidia. In contrast, Isp7-mCherry fluoresced most strongly at the basidial surface and within spores, with little fluorescence in other cell types. Interestingly, Isp1 deletion strains show a strong phenotype during early filamentation, but fluorescent protein was not visible during this stage of development in wild type strains.
(TIFF)

S3 Fig Yeast growth on YPD solid medium at room temperature (RT) or 37°C. Yeast of the same starting concentration were spotted at 10-fold serial dilutions and grown for 3 days at RT or 37°C.
(TIFF)

S4 Fig Yeast doubling times in YPD liquid culture. Doubling times (h) were calculated based on growth curves at 30°C. Five mutants, *isp1Δ*, *rsc9Δ*, *top1Δ*, *bch1Δ*, and *dst1Δ* showed significantly slower growth (* indicates p<0.05, ** indicates p<0.005, *** indicates p<0.001). Data represent 5 independent experiments and are shown as mean ± SD. An unpaired two-sided Student's t-test was used to assess significance.
(TIFF)

S5 Fig Further characterization of the filamentation defects of *rsc9Δ*, *isp1Δ*, and *bch1Δ* strains. (A) *isp1Δ* and *bch1Δ* mutants show defects after fusion. Images were taken 42h after the start of sexual development with 1000× magnification. Cells were stained with Calcofluor White (blue) for the cell wall and Sytox Green (green) for the nuclei. White arrows indicate the positions of nuclei. (B) The filamentation defects of *rsc9Δ*, *isp1Δ*, and *bch1Δ* crosses did not improve over time. Crosses were examined and photographed after 5 or 6 days on V8 plates at room temperature in the dark. Scale bars, 50μm (200× magnification).
(TIFF)

S6 Fig Crosses between *ddi1Δ*, *dst1Δ*, and *top1Δ* strains showed apparent defects in nuclear migration. Images were taken 96h after the start of sexual development. Cells were stained with Calcofluor White (blue) for the cell wall and Sytox Green (green) for the nuclei.
(TIFF)

S7 Fig Characterization of spores from deletion mutants of spore-enriched proteins. (A) No differences exists between wild type (left) and mutant spores (right, *isp2Δ* spores as a representative example) in morphology or staining of surface carbohydrates. Spores were stained with Calcofluor White (bound to chitin in cell wall with blue fluorescence) and Concanavalin A conjugated with FITC (FITC-ConA, bound to α-mannose residues on the spore surface with green fluorescence). Scale bars, 5μm (1000× magnification). (B) Spore viability on rich YPD medium was evaluated after treatment under several stress conditions as listed. (C) Different conditions were used to assess germination of mutant spores. Under all conditions, no significant differences were observed between wild type and mutant strains.
(TIFF)

S8 Fig Deletion of *ISP2* (CNE01730) does not affect the expression its neighboring genes. (A) A schematic of the genomic locus of *ISP2* (CNE01730) and its neighboring genes in wild type (WT), *isp2Δ*, and *ISP2* complementation strains. *ISP2* is located on chromosome 5 and the region shown is from 474,000 to 482,000. Each gene is represented by a gray or hatched bar pointing in the direction of transcription. *ISP2* (1281bp ORF) is 287bp downstream of CNE01720 (1800bp ORF) and 181bp upstream of CNE01740 (3496bp ORF). The entire *ISP2* ORF was replaced with a *URA5* marker (1768bp) (black bar) to create the *isp2Δ* strain. The *ISP2* complementation fragment contained the complete *ISP2* ORF, 577bp of upstream sequence, 380bp of downstream sequence, and a nourseothricin resistance (*NAT* ^*R*^) marker (white pointed bar) and was integrated randomly into the genome. (B) Transcript levels of the genes upstream and downstream of *ISP2* in the *isp2Δ* strain were indistinguishable from the wild type strain. Wild type and *isp2Δ* crosses were initiated on V8 plates and incubated for 72h before RNA extraction. Cross conditions were chosen for transcript analysis to ensure that sufficient RNA could be extracted and transcripts could be detected. qRT-PCR analysis was then performed to determine the relative expression levels of CNE01720, *ISP2* (CNE01730), and CNE01740. (C) Strains transformed with the complementation construct did not express *ISP2* at appreciable levels. In each case wild type, *isp2Δ*, and complemented crosses were initiated on V8 plates and incubated for 48h before RNA extraction. qRT-PCR analysis was then performed to determine the relative expression level of *ISP2* (CNE01730). In both B and C, the expression level of each gene is normalized to the internal reference gene *GPD1* and relative to wild type. Data represent 3 replicates and are shown as mean ± SD.
(TIFF)

S1 Table All protein identified in spores and yeast. (XLS)

S2 Table Proteins identified in previous studies. (DOC)

S3 Table Proteins of interest. (XLS)

S4 Table Spectral counts. (DOC)

S5 Table Strains and PCR primers used in this study. (DOC)
