# Supplementary figures and images for: Protein Composition of Infectious Spores Reveals Novel Sexual Development and Germination Factors in Cryptococcus
Source: PLoS Genet. 2015 Aug 27;11(8):e1005490. doi: 10.1371/journal.pgen.1005490 (PMC4551743; doi:10.1371/journal.pgen.1005490)

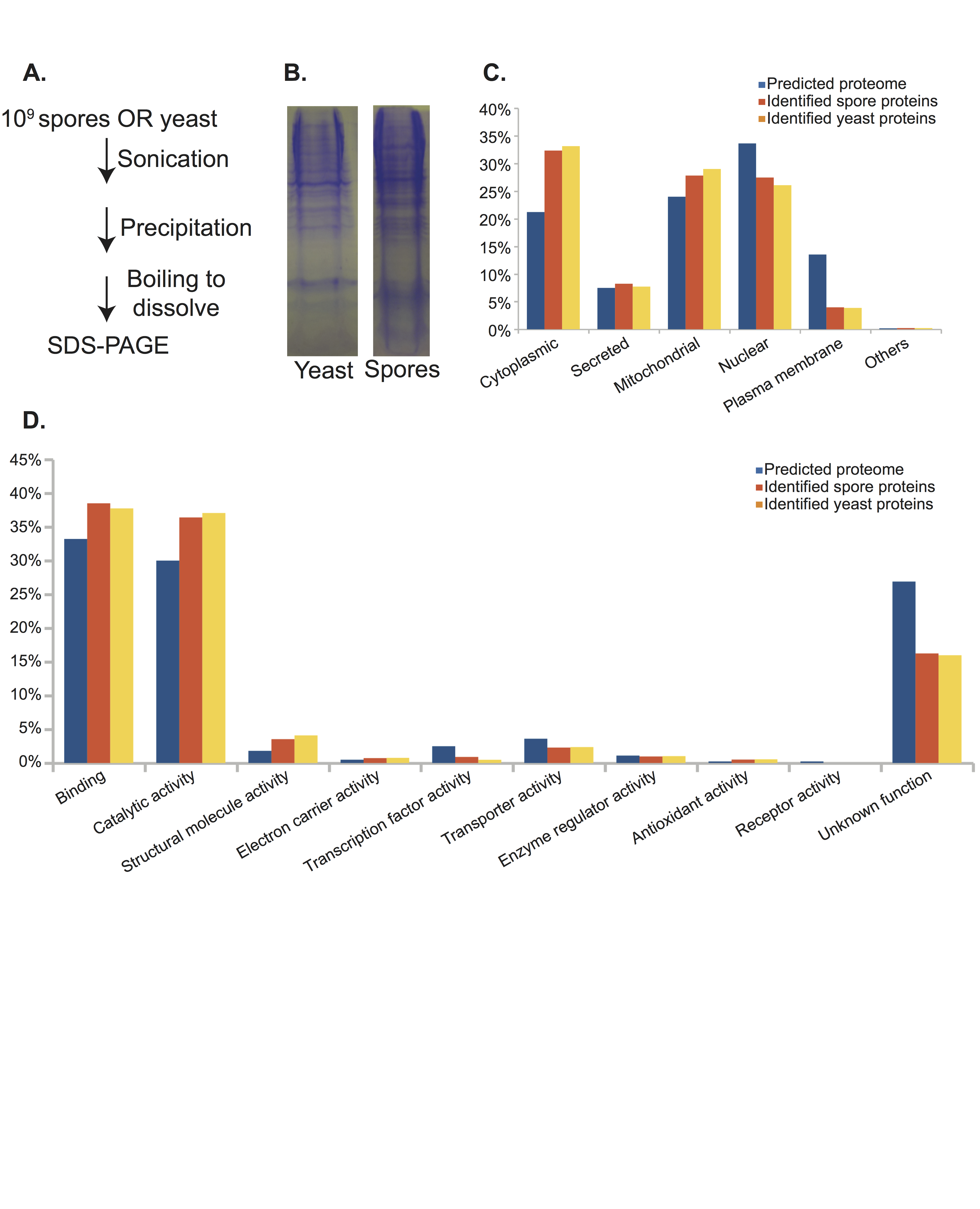

Supplement: S1 Fig — (A) Schematic workflow of protein exaction. A density-gradient centrifugation method was used to purify large quantities of spores from crosses between a and α cells. 1×10^9^ spores were sonicated to release proteins, which were subsequently precipitated by organic solvent. The resulting pellet was boiled in SDS-containing buffer to dissolve protein for SDS-PAGE analysis. (B) Protein extracts from yeast and spores were analyzed by SDS-PAGE. Coomassie Brilliant Blue-staining showed that sufficient amount of proteins (about 100μg estimated by staining) were obtained. (C) Distribution of subcellular localization for all the proteins encoded by the genome, identified in spores or yeast. Their localizations were predicted using WoLF PSORT. Plasma membrane proteins are under-represented and cytoplasmic proteins are correspondingly over-represented in our dataset of identified spore or yeast proteins. (D) Distribution of general molecular function for all the proteins encoded by the genome, identified in spores or yeast. Molecular functions were predicted and analyzed using Blast2GO. (TIFF) [file pgen.1005490.s001.tiff]

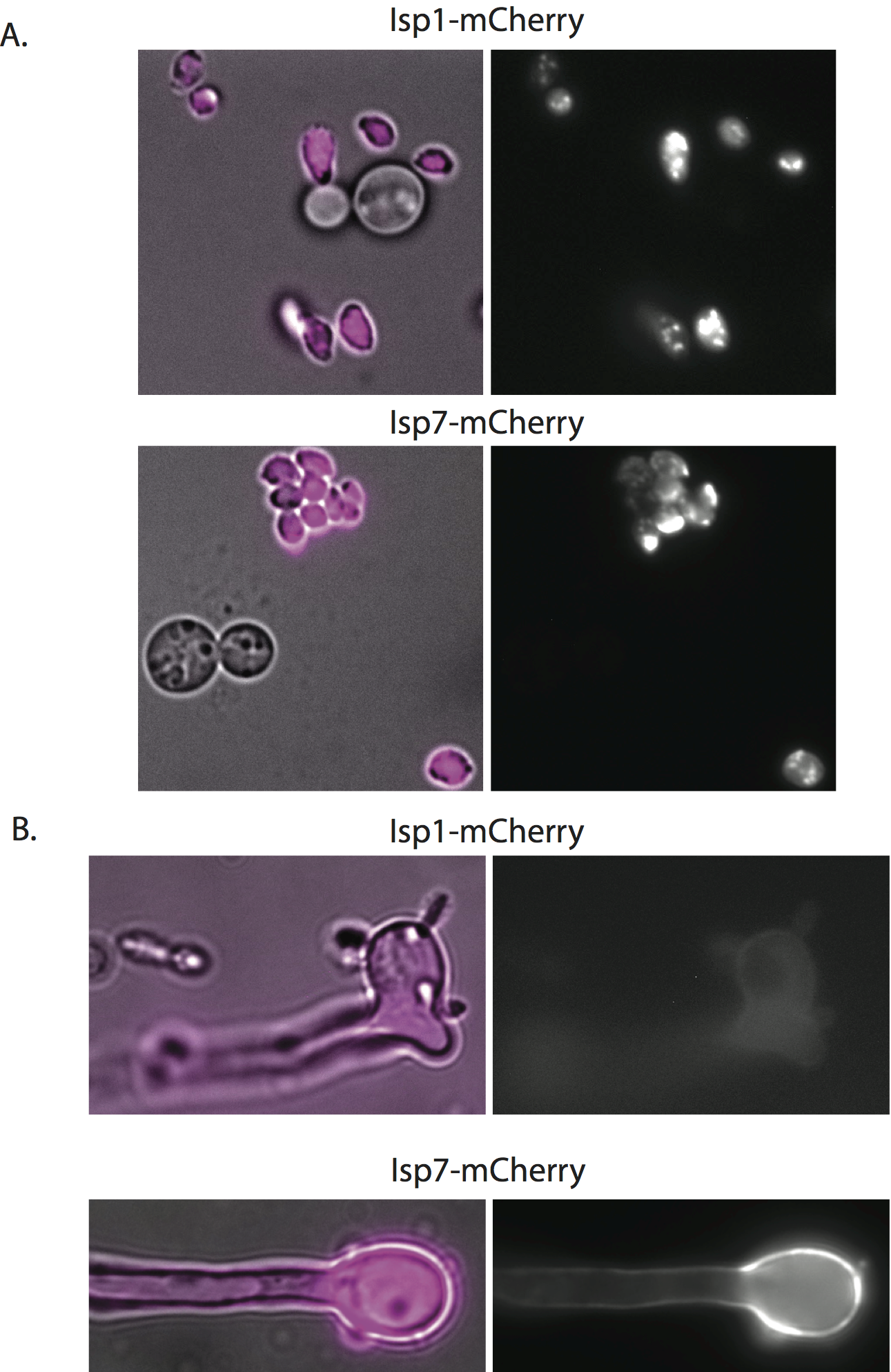

Supplement: S2 Fig — mCherry was fused to the C-termini of Isp1 and Isp7 and expressed under their endogenous promoters. (A) Spores and yeast or (B) basidia harboring each recombinant expression construct were visualized using fluorescence microscopy and photographed. Left panels are merged images of light field and red fluorescence channel. Right panels are images from the red fluorescence channel only. Isp1-mCherry, showed visible fluorescence in spores, but no levels of fluorescence over background in other cell types, including basidia. In contrast, Isp7-mCherry fluoresced most strongly at the basidial surface and within spores, with little fluorescence in other cell types. Interestingly, Isp1 deletion strains show a strong phenotype during early filamentation, but fluorescent protein was not visible during this stage of development in wild type strains. (TIFF) [file pgen.1005490.s002.tiff]

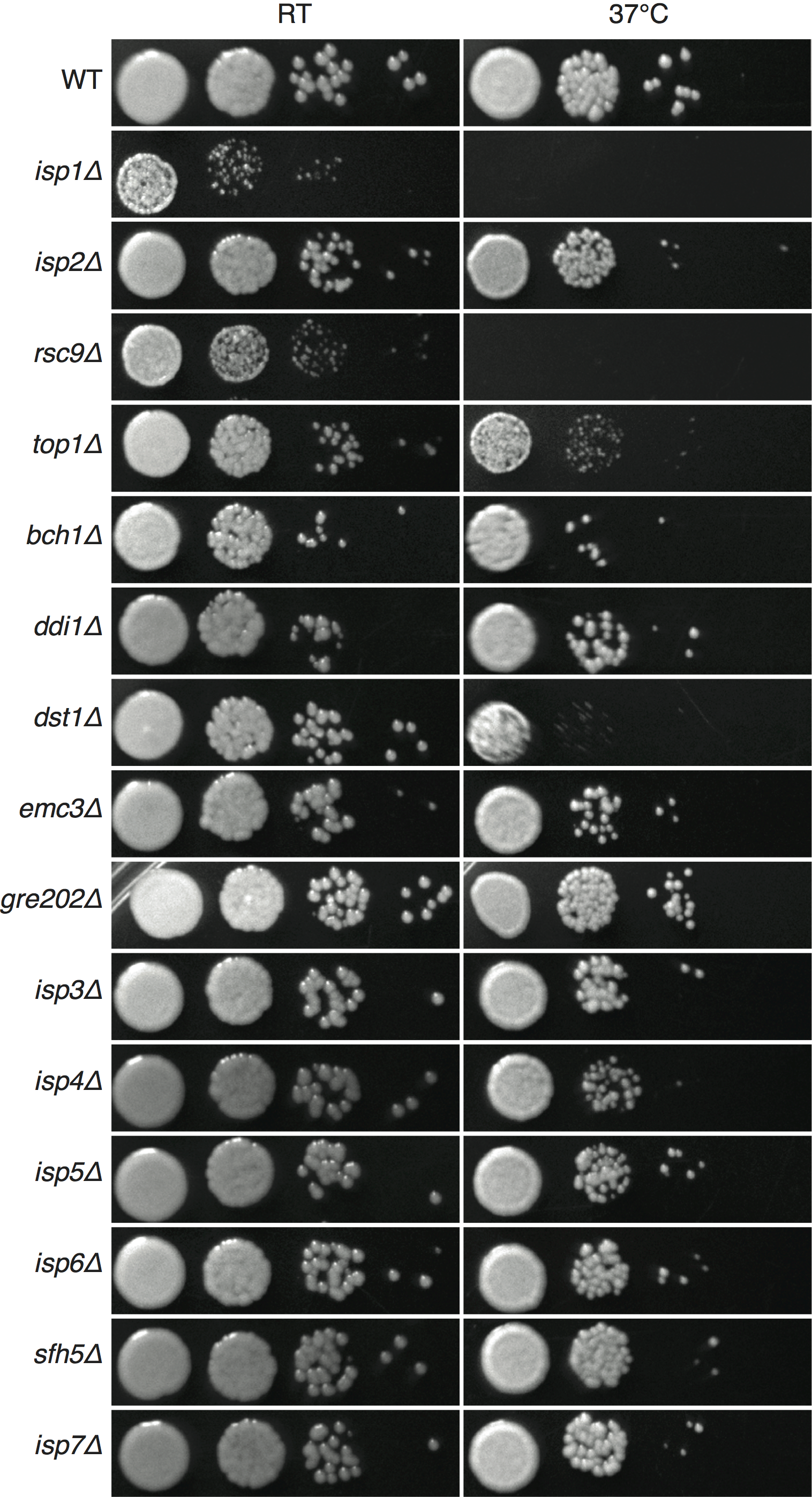

Supplement: S3 Fig — Yeast of the same starting concentration were spotted at 10-fold serial dilutions and grown for 3 days at RT or 37°C. (TIFF) [file pgen.1005490.s003.tiff]

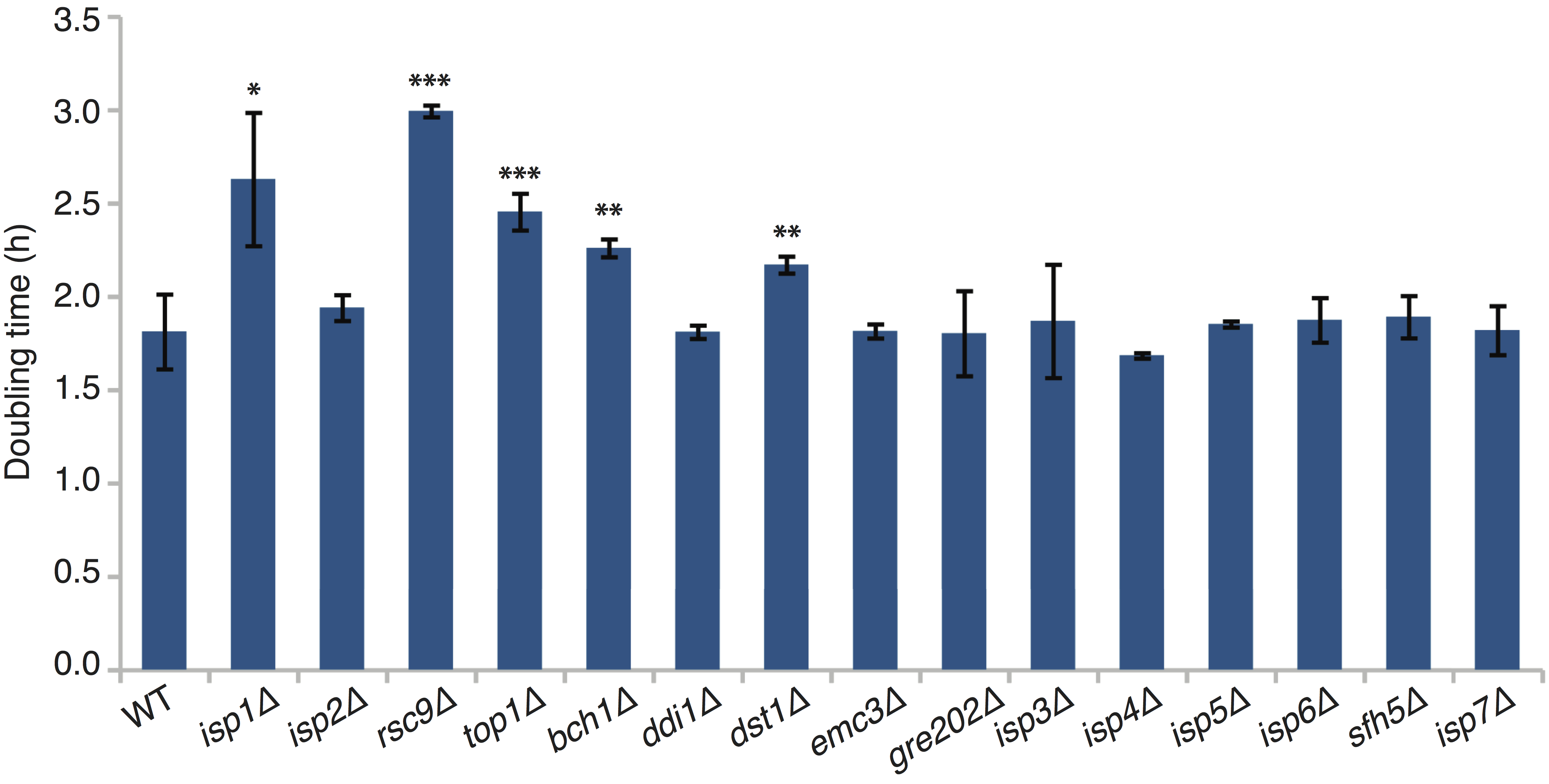

Supplement: S4 Fig — Doubling times (h) were calculated based on growth curves at 30°C. Five mutants, isp1Δ, rsc9Δ, top1Δ, bch1Δ, and dst1Δ showed significantly slower growth (* indicates p<0.05, ** indicates p<0.005, *** indicates p<0.001). Data represent 5 independent experiments and are shown as mean ± SD. An unpaired two-sided Student's t-test was used to assess significance. (TIFF) [file pgen.1005490.s004.tiff]

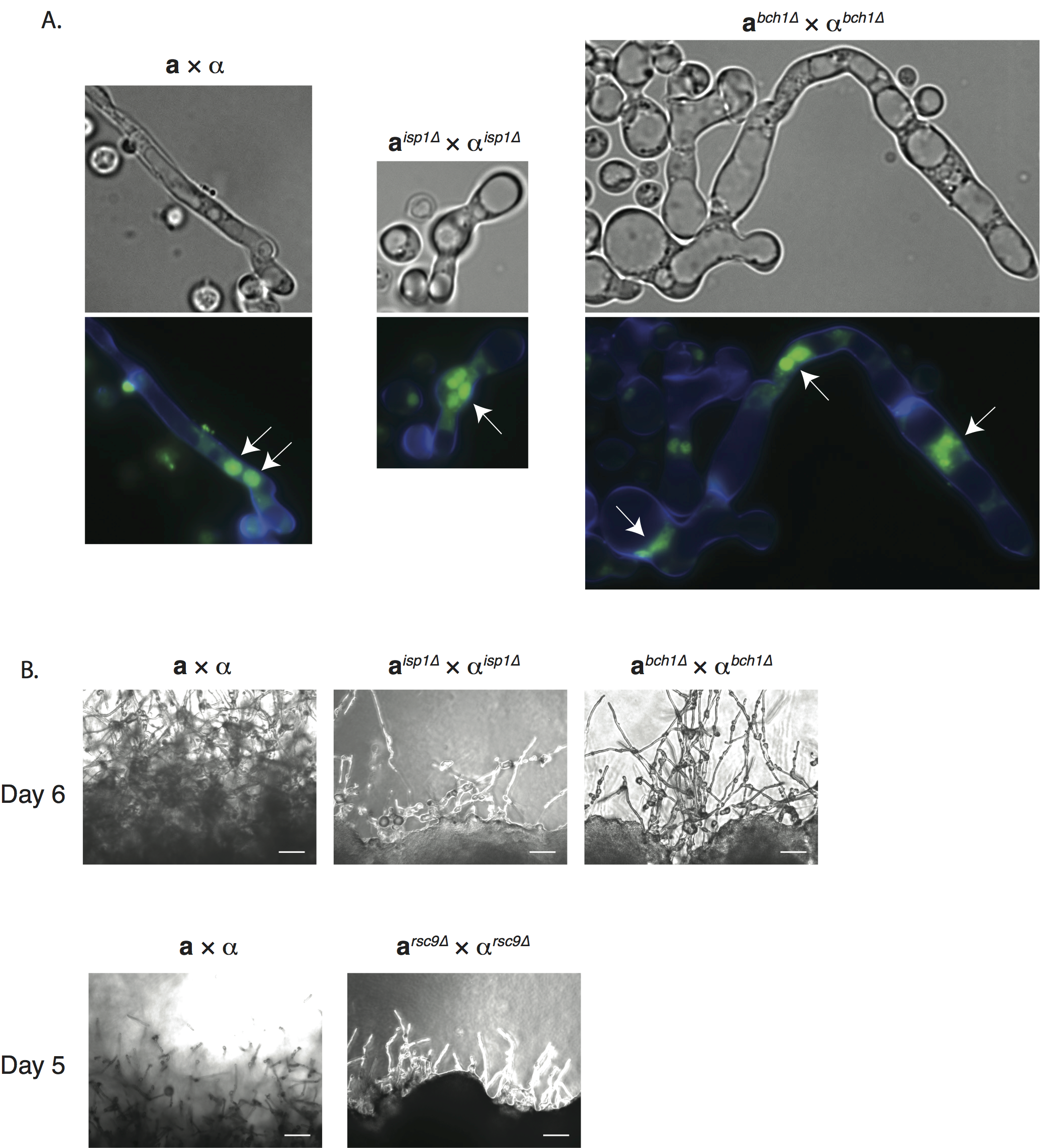

Supplement: S5 Fig — (A) isp1Δ and bch1Δ mutants show defects after fusion. Images were taken 42h after the start of sexual development with 1000× magnification. Cells were stained with Calcofluor White (blue) for the cell wall and Sytox Green (green) for the nuclei. White arrows indicate the positions of nuclei. (B) The filamentation defects of rsc9Δ, isp1Δ, and bch1Δ crosses did not improve over time. Crosses were examined and photographed after 5 or 6 days on V8 plates at room temperature in the dark. Scale bars, 50μm (200× magnification). (TIFF) [file pgen.1005490.s005.tiff]

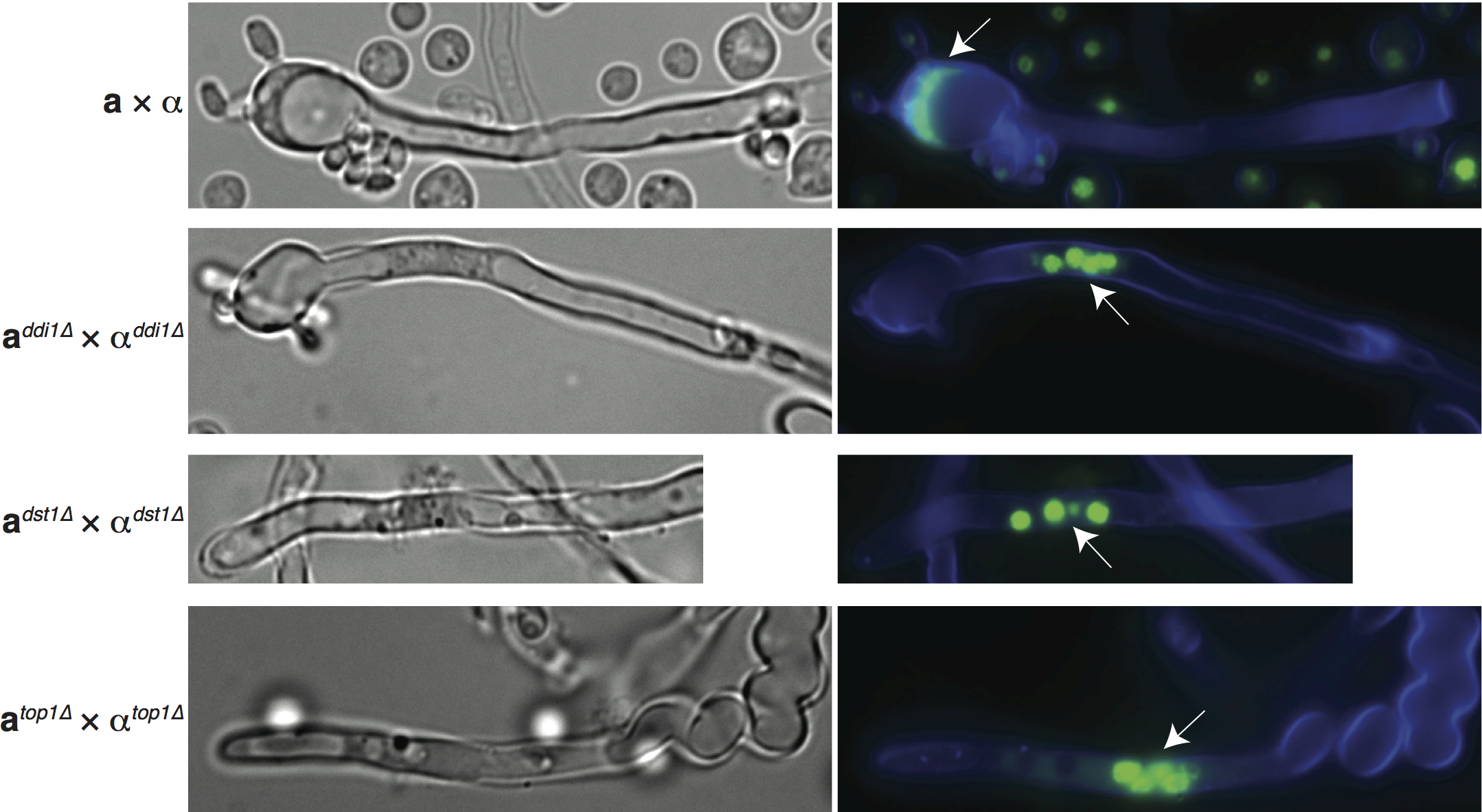

Supplement: S6 Fig — Images were taken 96h after the start of sexual development. Cells were stained with Calcofluor White (blue) for the cell wall and Sytox Green (green) for the nuclei. (TIFF) [file pgen.1005490.s006.tiff]

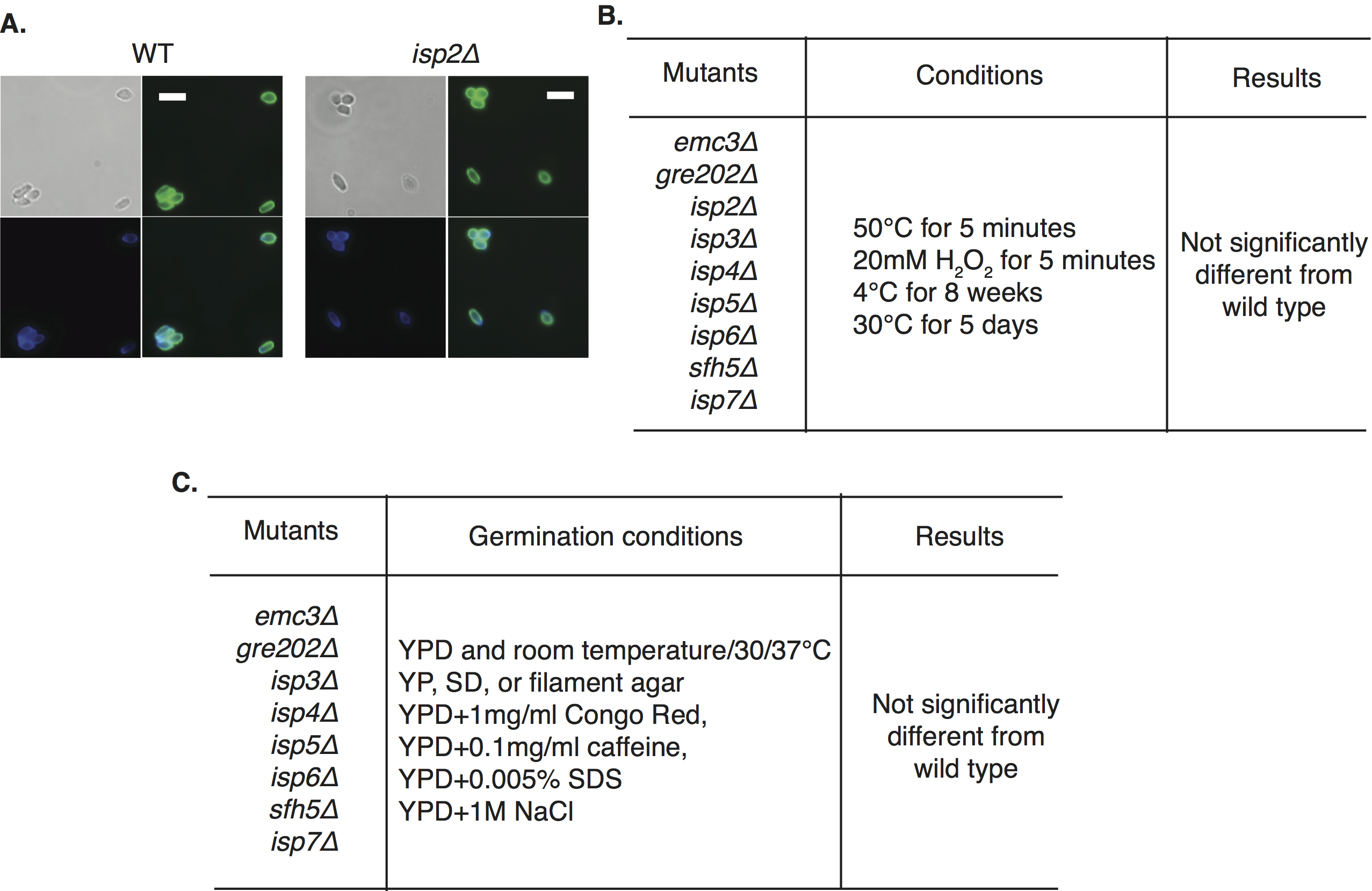

Supplement: S7 Fig — (A) No differences exists between wild type (left) and mutant spores (right, isp2Δ spores as a representative example) in morphology or staining of surface carbohydrates. Spores were stained with Calcofluor White (bound to chitin in cell wall with blue fluorescence) and Concanavalin A conjugated with FITC (FITC-ConA, bound to α-mannose residues on the spore surface with green fluorescence). Scale bars, 5μm (1000× magnification). (B) Spore viability on rich YPD medium was evaluated after treatment under several stress conditions as listed. (C) Different conditions were used to assess germination of mutant spores. Under all conditions, no significant differences were observed between wild type and mutant strains. (TIFF) [file pgen.1005490.s007.tiff]

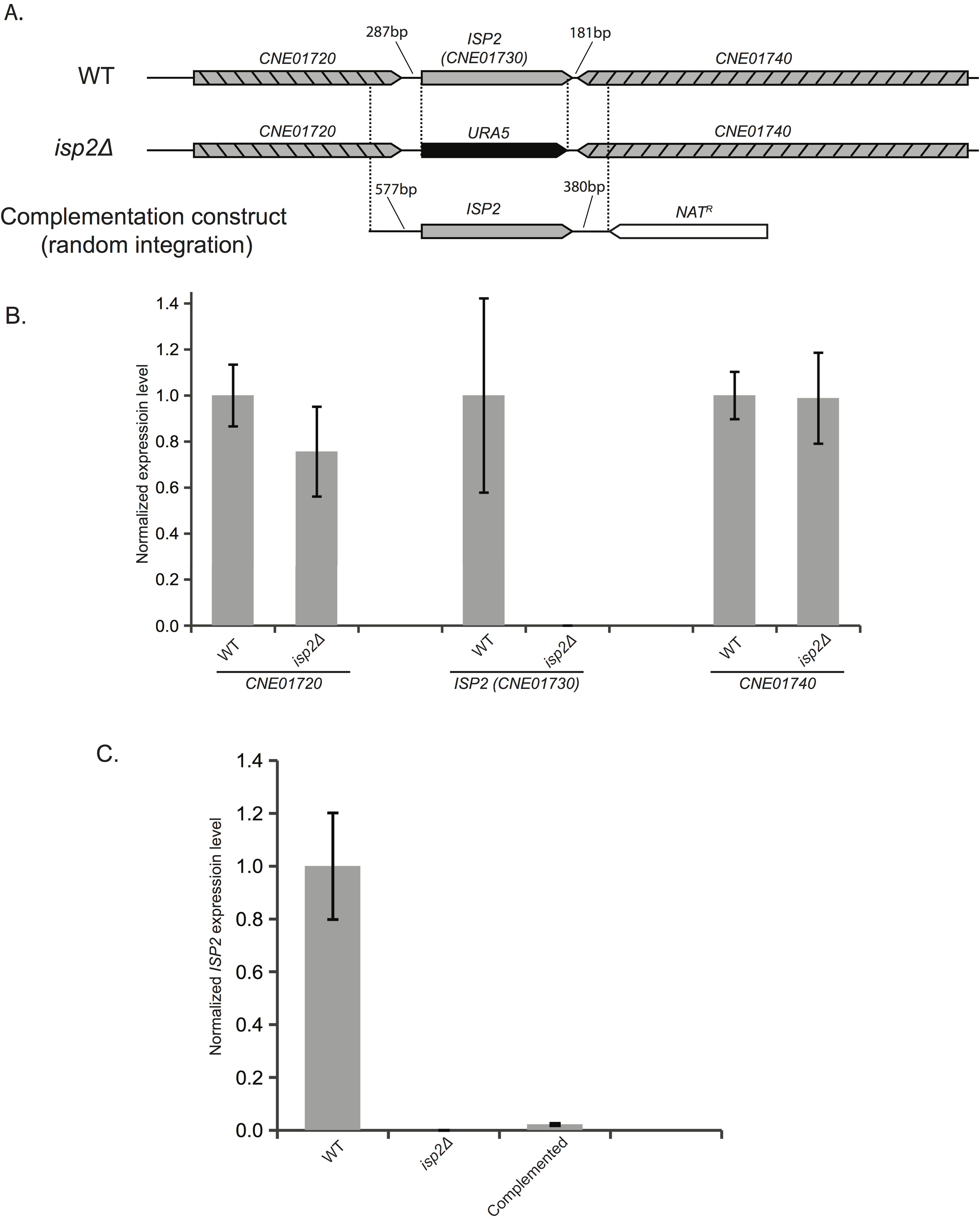

Supplement: S8 Fig — (A) A schematic of the genomic locus of ISP2 (CNE01730) and its neighboring genes in wild type (WT), isp2Δ, and ISP2 complementation strains. ISP2 is located on chromosome 5 and the region shown is from 474,000 to 482,000. Each gene is represented by a gray or hatched bar pointing in the direction of transcription. ISP2 (1281bp ORF) is 287bp downstream of CNE01720 (1800bp ORF) and 181bp upstream of CNE01740 (3496bp ORF). The entire ISP2 ORF was replaced with a URA5 marker (1768bp) (black bar) to create the isp2Δ strain. The ISP2 complementation fragment contained the complete ISP2 ORF, 577bp of upstream sequence, 380bp of downstream sequence, and a nourseothricin resistance (NAT R) marker (white pointed bar) and was integrated randomly into the genome. (B) Transcript levels of the genes upstream and downstream of ISP2 in the isp2Δ strain were indistinguishable from the wild type strain. Wild type and isp2Δ crosses were initiated on V8 plates and incubated for 72h before RNA extraction. Cross conditions were chosen for transcript analysis to ensure that sufficient RNA could be extracted and transcripts could be detected. qRT-PCR analysis was then performed to determine the relative expression levels of CNE01720, ISP2 (CNE01730), and CNE01740. (C) Strains transformed with the complementation construct did not express ISP2 at appreciable levels. In each case wild type, isp2Δ, and complemented crosses were initiated on V8 plates and incubated for 48h before RNA extraction. qRT-PCR analysis was then performed to determine the relative expression level of ISP2 (CNE01730). In both B and C, the expression level of each gene is normalized to the internal reference gene GPD1 and relative to wild type. Data represent 3 replicates and are shown as mean ± SD. (TIFF) [file pgen.1005490.s008.tiff]
