# Supplementary material for: Protein Composition of Infectious Spores Reveals Novel Sexual Development and Germination Factors in Cryptococcus
Source: PLoS Genet. 2015 Aug 27;11(8):e1005490. doi: 10.1371/journal.pgen.1005490 (PMC4551743; doi:10.1371/journal.pgen.1005490)
Supplement: S2 Table — (DOC) [file pgen.1005490.s010.doc]

**S2 Table. Proteins identified in previous studies**

| **Accession number** | **Protein function** | **References** | **Identification in the present dataset** |
| --- | --- | --- | --- |
| CNBE3490 | Alpha-Amylase | [20] | Yes |
| CNBG4090 | Trehalase | [20] | Yes |
| CNBC2400 | Endo-1,3-b-glucanase | [20] | Yes |
| CNBM0110 | MP88-like | [20] | Yes |
| CNBA7360 | MP88 | [20] | Yes |
| CNBD2840 | Chitin deacetylase 2 | [20] | Yes |
| CNBD2750 | Chitin deacetylase 3 | [20] | Yes |
| CNBF2910 | Chitin deactylase 1 | [20] | Yes |
| CNBN0250 | Polysaccharide deacetylase | [20] | Yes |
| CNBC1420 | Serine protease 1 | [20] | Yes |
| CNBF4600 | Serine protease 2 | [20] | Yes |
| CNBH1590 | Aspartic protease | [20] | Yes |
| CNBA5450 | Aspartic protease | [20] | Yes |
| CNBA1340 | Serine protease 1 (subtilase family) | [20] | Yes |
| CNBJ2870 | Serine protease 2 (subtilase family) | [20] | Yes |
| CNBJ1810 | Metalloprotease | [20] | Yes |
| CNBJ0550 | Carboxylesterase 1 | [20] | Yes |
| CNBC0510 | Carboxylesterase 2 | [20] | Yes |
| CNBE5040 | Glyoxal oxidase 1 | [20] | Yes |
| CNBA3760 | Glyoxal oxidase 3 | [20] | Yes |
| CNBN2300 | Gas1 | [20] | Yes |
| CNBD4830 | Superoxide dismutase 152 | [20] | Yes |
| CNBD4370 | PG-PI transfer protein | [20] | Yes |
| CNBI2180 | CFEM domain, fungal specific cysteine rich domain | [20] | No |
| CNBC3230 | Unknown | [20] | Yes |
| CNBG1270 | Unknown | [20] | No |
| CNBL2400 | Kelch motif, related to Galactose Oxidase | [20] | Yes |
| CNBG1100 | Unknown | [20] | No |
| CNBE4750 | Unknown | [20] | No |
| CNBC6500 | Lipoprotein | [20] | Yes |
| CNK03170 | Transaldolase | [21] | Yes |
| CNF00400 | UMP-CMP kinase | [21] | Yes |
| CNA05850 | Hypothetical protein | [21] | Yes |
| CNC00160 | Phosphopyruvate hydratase (enolase) | [21] | Yes |
| CND06290 | Saccharopine dehydrogenase | [21] | Yes |
| CNA07620 | Translation initiation factor | [21] | Yes |
| CNC02320 | Heat shock protein 70 | [21] | Yes |
| CND00940 | Hypothetical protein | [21] | Yes |
| CNK02370 | Aldehyde reductase i | [21] | Yes |
| CNL05250 | Cytoplasm protein | [21] | Yes |
| CNE03540 | D-lactaldehyde dehydrogenase | [21] | Yes |
| CNB00990 | 2-oxoglutarate metabolism related protein | [21] | Yes |
| CNM01520 | Heat shock protein 90 | [21] | Yes |
| CNB00990 | 2-oxoglutarate metabolism related protein | [21] | Yes |
| CNL03930 | 14-3-3 protein | [21] | Yes |

a. 25 out of 29 *C. neoformans* secreted and cell wall-bound proteins proteins were identified in current study [20].

b. 15 out of 15 *C. neoformans* immunodominant proteins were identified in current study [21].
