# Supplementary material for: Protein Composition of Infectious Spores Reveals Novel Sexual Development and Germination Factors in Cryptococcus
Source: PLoS Genet. 2015 Aug 27;11(8):e1005490. doi: 10.1371/journal.pgen.1005490 (PMC4551743; doi:10.1371/journal.pgen.1005490)
Supplement: S4 Table — (DOC) [file pgen.1005490.s012.doc]

**S4 Table. Spectral counts in three replicates of shotgun proteomic experiments**

| **Spectral counts** | **Replicate 1** | | **Replicate 2** | | **Replicate 3** | |
| --- | --- | --- | --- | --- | --- | --- |
|  | **Total** | **Average** | **Total** | **Average** | **Total** | **Average** |
| **Spore** | 14739 | 3.46 | 9931 | 2.22 | 13810 | 3.18 |
| **Yeast** | 16792 | 4.19 | 15921 | 3.30 | 19172 | 4.15 |

a. The global average numbers of peptides detected per protein per experiment were calculated excluding the top and bottom fifth percentile data to prevent skewing.
