# Supplementary material for: Protein Composition of Infectious Spores Reveals Novel Sexual Development and Germination Factors in Cryptococcus
Source: PLoS Genet. 2015 Aug 27;11(8):e1005490. doi: 10.1371/journal.pgen.1005490 (PMC4551743; doi:10.1371/journal.pgen.1005490)
Supplement: S5 Table — (DOC) [file pgen.1005490.s013.doc]

**S5 Table. Strains and PCR primers used in this study**

| **Strain** | **Genotype** | **Reference** |
| --- | --- | --- |
| JEC20 | **a** | [56] |
| JEC21 | α | [56] |
| JEC34 | **a** *ura* | [57] |
| JEC43 | *α ura* | [57] |
| CHY3646-3648 | ***a*** *rsc9Δ::URA5* | This study |
| CHY3643-3645 | *α rsc9Δ::URA5* | This study |
| CHY3612-3614 | ***a*** *isp1Δ::URA5* | This study |
| CHY3615-3617 | *α isp1Δ::URA5* | This study |
| CHY3673-3675 | ***a*** *bch1Δ::URA5* | This study |
| CHY3676-3678 | *α bch1Δ::URA5* | This study |
| CHY3606-3608 | ***a*** *ddi1Δ::URA5* | This study |
| CHY3609-3611 | *α ddi1Δ::URA5* | This study |
| CHY3679-3681 | ***a*** *dst1Δ::URA5* | This study |
| CHY3682-3684 | *α dst1Δ::URA5* | This study |
| CHY3649-3651 | ***a*** *top1Δ::URA5* | This study |
| CHY3652-3654 | *α top1Δ::URA5* | This study |
| CHY3661-3663 | ***a*** *emc3Δ::URA5* | This study |
| CHY3664-3666 | *α emc3Δ::URA5* | This study |
| CHY3667-3669 | ***a*** *gre202Δ::URA5* | This study |
| CHY3670-3672 | *α gre202Δ::URA5* | This study |
| CHY3655-3657 | ***a*** *isp2Δ::URA5* | This study |
| CHY3658-3660 | *α isp2Δ::URA5* | This study |
| CHY3602-3604 | ***a****, isp3Δ::NEO^R^* | This study |
| CHY3605 | *α, isp3Δ::NEO^R^* | This study |
| CHY3621-3622 | ***a****, isp4Δ::NAT^R^* | This study |
| CHY3618-3620 | *α, isp4Δ::NAT^R^* | This study |
| CHY3631-3633 | ***a*** *isp5Δ::URA5* | This study |
| CHY3634-3636 | *α isp5Δ::URA5* | This study |
| CHY3637-3639 | ***a*** *isp6Δ::URA5* | This study |
| CHY3640-3642 | *α isp6Δ::URA5* | This study |
| CHY3629 | ***a****, sfh5Δ::NEO^R^* | This study |
| CHY3630 | *α, sfh5Δ::NEO^R^* | This study |
| CHY3626-3628 | ***a****, isp7Δ::NAT^R^* | This study |
| CHY3623-3625 | *α, isp7Δ::NAT^R^* | This study |

**Primers for gene knock-out, screening and qRT-PCR:**

| **Descriptions** | **Primers** |
| --- | --- |
| *irr1Δ::URA5* | P1, CHO4793: CAACGTACCCAAGTCTCCCC  P2, CHO4794: GGTCGAGCAACTTCGCTCCGGCAATGAAGCGACAGAAG  P3, CHO4795: CTTCTGTCGCTTCATTGCCGGAGCGAAGTTGCTCGACC  P4, CHO4796: AGAACAAAACGAGCGTGTGCCTTGCCTCCAGGAGGTGG  P5, CHO4797: CCACCTCCTGGAGGCAAGGCACACGCTCGTTTTGTTCT  P6, CHO4798: GAGGCTCTGATTGCAGTGGT  P7, CHO4799: GGATCGACCGGGAAACGCAC  P8, CHO4800: ACCGTTTCGGAGTATCGCAT  P9, CHO4801: CAGAGGAGCAGTTGGTTCGT  P10, CHO4802: GACGGCTTGATGAGTGGCTA |
| *prp31Δ::URA5* | P1, CHO4803: CCCACGCCCTAAACTTCCTT  P2, CHO4804: GGTCGAGCAACTTCGCTCGGTGATGTGAGAGACCTGGC  P3, CHO4805: GCCAGGTCTCTCACATCACCGAGCGAAGTTGCTCGACC  P4, CHO4806: ACACCATTCGTGACTGTCCCCTTGCCTCCAGGAGGTGG  P5, CHO4807: CCACCTCCTGGAGGCAAGGGGACAGTCACGAATGGTGT  P6, CHO4808: CTCCAGAGGATAGCGCACAG  P7, CHO4809: CCTCTTGTTCGTTCGCGGCT  P8, CHO4810: AGGTGAGCTTTTGAGCCCTT  P9, CHO4811: GCGTGGTGGTAAGAGGTCAG  P10, CHO4812: GGATGTGACTGAGCGACCAA |
| *prp11Δ::URA5* | P1, CHO4890: ACCCCGTCAGTTTTTCCAGT  P2, CHO4891: GGTCGAGCAACTTCGCTCAGCATTTGCCATCCCCTCAA  P3, CHO4892: TTGAGGGGATGGCAAATGCTGAGCGAAGTTGCTCGACC  P4, CHO4893: AACGTACTGACCCGTTTGCACTTGCCTCCAGGAGGTGG  P5, CHO4894: CCACCTCCTGGAGGCAAGTGCAAACGGGTCAGTACGTT  P6, CHO4895: CGTAAGGTTTCCCTCCCCAC  P7, CHO4896: GGCATTGGACGCTAGGGAGC  P8, CHO4897: GTGGAAGGCGTTGAGTACAG  P9, CHO4898: CGCCGTTGTTCAAAAGCAGA  P10, CHO4899: GAGTCTGATTCACGGCCCAG |
| *rsc9Δ::URA5* | P1, CHO4604: GATGACTAACAGCCACGCCT  P2, CHO4823: GGTCGAGCAACTTCGCTCTGCAAAGCTCTCACAACGGA  P3, CHO4824: TCCGTTGTGAGAGCTTTGCAGAGCGAAGTTGCTCGACC  P4, CHO4825: AAAGCGAGGAAGCTTGAGCTCTTGCCTCCAGGAGGTGG  P5, CHO4826: CCACCTCCTGGAGGCAAGAGCTCAAGCTTCCTCGCTTT  P6, CHO4609: TGCACGTACCTGATCGCTTC  P7, CHO4751: CCGACTCATCCTTCCGTGCGT  P8, CHO4752: GCTTTCTACAGCCCAAGCGCA  P9, CHO4749: CCGTTGTGAGAGCTTTGCAT  P10, CHO4750: CCATTCCTTCAACGCGCAAA |
| *isp1Δ::URA5* | P1, CHO4860: GCACGGGGTTTCAAGACCTA  P2, CHO4861: GGTCGAGCAACTTCGCTCTAGCGCTTTCCCAGTTCGAG  P3, CHO4862: CTCGAACTGGGAAAGCGCTAGAGCGAAGTTGCTCGACC  P4, CHO4863: TCAACCAGACGTGGAAGACGCTTGCCTCCAGGAGGTGG  P5, CHO4864: CCACCTCCTGGAGGCAAGCGTCTTCCACGTCTGGTTGA  P6, CHO4865: ACTCTGCTCTTGCCTTCGAC  P7, CHO4866: AGTCCATGTCCGCCCCAAGA  P8, CHO4867: ATGTCGCATATCAAGCCGTT  P9, CHO4868: TCTGGTGATCAGCGAGTCGT  P10, CHO4869: CCCGTCGTGAAAGGAAGTGA |
| *bch1Δ::URA5* | P1, CHO4930: GTGGCCTGAAGGTAGCATGA  P2, CHO4931: GGTCGAGCAACTTCGCTCATGAAATACGGGGCGACGTT  P3, CHO4932: AACGTCGCCCCGTATTTCATGAGCGAAGTTGCTCGACC  P4, CHO4933: GAGTATGCCTCTGGCGAGACCTTGCCTCCAGGAGGTGG  P5, CHO4934: CCACCTCCTGGAGGCAAGGTCTCGCCAGAGGCATACTC  P6, CHO4935: TTCGACTTCCTTCACAGCCC  P7, CHO4936: TCCAGCTAACAGGCCAGGGT  P8, CHO4937: GAGCTTTGATTGTGAGGCCG  P9, CHO4938: GACCGGCAGGTTCTGTCATT  P10, CHO4939: CTTGGTGAACTTGCCACACG |
| *ddi1Δ::URA5* | P1, CHO4696: CGAAAGGCCTCCATTCAGGT  P2, CHO4697: GGTCGAGCAACTTCGCTCCCGAGGACGACTCACAAACA  P3, CHO4698: TGTTTGTGAGTCGTCCTCGGGAGCGAAGTTGCTCGACC  P4, CHO4699: CGTGGAGAGGTCGATACAGCCTTGCCTCCAGGAGGTGG  P5, CHO4700: CCACCTCCTGGAGGCAAGGCTGTATCGACCTCTCCACG  P6, CHO4701: TCTTCTTGCGTTCCCTGACC  P7, CHO4720: CCTGCTCGGGGCGAAAAGAC  P8, CHO4721: GTTTTGACGGAAACGCGTGA  P9, CHO4722: AAGACGTGGAAGATAGCGCC  P10, CHO4723: ACAATAACAGCCGGGGGAAG |
| *dst1Δ::URA5* | P1, CHO4813: ACACCGTGCTGTATCTTGGG  P2, CHO4814: GGTCGAGCAACTTCGCTCTTGCCAAGGTGAGCGAGAAA  P3, CHO4815: TTTCTCGCTCACCTTGGCAAGAGCGAAGTTGCTCGACC  P4, CHO4816: CAAAAACGCCGGTTCCGTTTCTTGCCTCCAGGAGGTGG  P5, CHO4817: CCACCTCCTGGAGGCAAGAAACGGAACCGGCGTTTTTG  P6, CHO4818: GGTCGGCTCAACAATCCCTT  P7, CHO4819: CCAGGAGTGCCAAGAGGGGT  P8, CHO4820: CAGAAAGGGGACTGACCTCG  P9, CHO4821: TGCGTCGTTCTTTCGTAGGT  P10, CHO4822: TGCACACATACTTTCCGCCT |
| *top1Δ::URA5* | P1, CHO4850: CTTTTGCTGCTAACATCGCCT  P2, CHO4851: GGTCGAGCAACTTCGCTCCAGGTGTTTAGCCATTGCCG  P3, CHO4852: CGGCAATGGCTAAACACCTGGAGCGAAGTTGCTCGACC  P4, CHO4853: ACTGTACCGCCCAACGTAAACTTGCCTCCAGGAGGTGG  P5, CHO4854: CCACCTCCTGGAGGCAAGTTTACGTTGGGCGGTACAGT  P6, CHO4855: GCCTCTCATACCATCCAGCC  P7, CHO4856: ACGTAGCAAGGTCCCAGCACT  P8, CHO4857: TTAGCCGGAATCTGCCCTAC  P9, CHO4858: ATCGCCTTCAAACTGACCGA  P10, CHO4859: TCTTCAGACTCGGGAGGGAG |
| *emc3Δ::URA5* | P1, CHO4910: TGCGAATTGCTGCATCTTCC  P2, CHO4911: GGTCGAGCAACTTCGCTCGGGACCGAGAGCAGGGATTA  P3, CHO4912: TAATCCCTGCTCTCGGTCCCGAGCGAAGTTGCTCGACC  P4, CHO4913: TTTAACGACCACCGTAGCCCCTTGCCTCCAGGAGGTGG  P5, CHO4914: CCACCTCCTGGAGGCAAGGGGCTACGGTGGTCGTTAAA  P6, CHO4915: GATACCAAGGGCAACAGCCT  P7, CHO4916: GCCCTGTACTTGGCCACTTCT  P8, CHO4917: TCGAAGGTGGTCTCGTAAAGG  P9, CHO4918: GCCTTGTTGTTGGGTGTTGG  P10, CHO4919: TGATCTCGATGTGCGATGGG |
| *gre202Δ::URA5* | P1, CHO4920: TTGCTTGCAATGAGTGGCTG  P2, CHO4921: GGTCGAGCAACTTCGCTCGCGCTCCAGCTTCCTATCTT  P3, CHO4922: AAGATAGGAAGCTGGAGCGCGAGCGAAGTTGCTCGACC  P4, CHO4923: CTTGCTATCTCGCGGACCTTCTTGCCTCCAGGAGGTGG  P5, CHO4924: CCACCTCCTGGAGGCAAGAAGGTCCGCGAGATAGCAAG  P6, CHO4925: TTGTTCTGCGCTTTGCACTC  P7, CHO4926: CCACCCGTTCCAGCAACCAA  P8, CHO4927: CGTAAGTTGTGAGCGCCAG  P9, CHO4928: TTGCGTACTTCGTATCGCGT  P10, CHO4929: CTGAGGACACCCCTTGTTCC |
| *isp2Δ::URA5* | P1, CHO4900: GTATGTCGTACCCGTCAGCG  P2, CHO4901: GGTCGAGCAACTTCGCTCGATTGGGCAACACACTTCCG  P3, CHO4902: CGGAAGTGTGTTGCCCAATCGAGCGAAGTTGCTCGACC  P4, CHO4903: CCCCGACACAAGAACCACAACTTGCCTCCAGGAGGTGG  P5, CHO4904: CCACCTCCTGGAGGCAAGTTGTGGTTCTTGTGTCGGGG  P6, CHO4905: AGGACGAAGGCCACTCAATG  P7, CHO4906: GGCGTAGACACCCAGCTCCT  P8, CHO4907: CTCAGACTTCAGTGCCGGTT  P9, CHO4908: CCTCTCCTTCTCCCATCCCA  P10, CHO4909: ACCGCTTGGCACTGACTTTA |
| *isp3Δ::NEO^R^* | P1, CHO4598: TATTACCCGGCCCACCACTA  P2, CHO4599: CAGCACACTGGCGGCCGTTATTGGCCACCTTTCCACAGTT  P3, CHO4600: AACTGTGGAAAGGTGGCCAATAACGGCCGCCAGTGTGCTG  P4, CHO4601: AACGACTCCATACTCACCCGAGCGTCACCGTTGGCACCAG  P5, CHO4602: CTGGTGCCAACGGTGACGCTCGGGTGAGTATGGAGTCGTT  P6, CHO4603: CCACTTCTTTGCCAATCGCC  P7, CHO4632: CTCGCCACCCACGCACTTCT  P8, CHO4633: AAGGCGGCTCGTGATTGGCT  P9, CHO4634: GGTCAGTTTCTTCCCGGCAT  P10, CHO4635: TTCAGAGTGGAAACGCCCTC |
| *isp4Δ::NAT^R^* | P1, CHO4610: ATGGTGGGCGAAGTCCAAAT  P2, CHO4611: AACCATACACTCAGCGCACATTCAGTCTAGGGCGCAACAG  P3, CHO4612: CTGTTGCGCCCTAGACTGAATGTGCGCTGAGTGTATGGTT  P4, CHO4613: TTTATCCTGCTGTGGCTGCATGTGCTGGAATTCGCCCTT  P5, CHO4614: AAGGGCGAATTCCAGCACATGCAGCCACAGCAGGATAAA  P6, CHO4615: ATCGCAAATGCATCGGCTTC  P7, CHO4645: GGCACCTCCGACAAAGAAGA  P8, CHO4646: GTCGAGTGACGTTGCTGCTA  P9, CHO4647: TGTGCGCTGAGTGTATGGTT  P10, CHO4648: AGCCTCGTGATGGACCCTAT |
| *isp5Δ::URA5* | P1, CHO4463: GACGCCAACAAGGAGCCCCG  P2, CHO4622: GGTCGAGCAACTTCGCTCACTTGGGCTGTCAAGACCTC  P3, CHO4623: GAGGTCTTGACAGCCCAAGTGAGCGAAGTTGCTCGACC  P4, CHO4624: GCTGGCGGAAACGTCAATACCTTGCCTCCAGGAGGTGG  P5, CHO4625: CCACCTCCTGGAGGCAAGGTATTGACGTTTCCGCCAGC  P6, CHO4468: TCGGATCTCGGGGGCGACAG  P7, CHO4702: TCACTTCTTCATCGGCAGCG  P8, CHO4703: CTCCACCACGGCCTTATCTT  P9, CHO4704: CACCGTCAACATGTGCGTTT  P10, CHO4705: CAAACCGAACCGACCAAACC |
| *isp6Δ::URA5* | P1, CHO4753: ACAGGATGACGCAATGGAGC  P2, CHO4754: GGTCGAGCAACTTCGCTCGCGTGGATTTAGTGGTTCGC  P3, CHO4755: GCGAACCACTAAATCCACGCGAGCGAAGTTGCTCGACC  P4, CHO4756: GCAGGCTTACCACTACCGTTCTTGCCTCCAGGAGGTGG  P5, CHO4757: CCACCTCCTGGAGGCAAGAACGGTAGTGGTAAGCCTGC  P6, CHO4758: TGCACTTAAACAGCCGCAAC  P7, CHO4789: TGGGTCGGAGAAGCTGAACG  P8, CHO4790: ACCTCTAGCTTCAGCCACAT  P9, CHO4791: GGGCCCGTATTGACGACTAC  P10, CHO4792: GGTAGAATGCCTCCTCCTGC |
| *sfh5Δ::NEO^R^* | P1, CHO4637: GAGTGCTGAGAGATCGACGG  P2, CHO4638: CAGCACACTGGCGGCCGTTATGCTCGCCAAGTGTTATCGT  P3, CHO4639: ACGATAACACTTGGCGAGCATAACGGCCGCCAGTGTGCTG  P4, CHO4640: TCTACTTGCTCAGCCAGACGAGCGTCACCGTTGGCACCAG  P5, CHO4641: CTGGTGCCAACGGTGACGCTCGTCTGGCTGAGCAAGTAGA  P6, CHO4642: GGAGGCGAGTATGAACGAGG  P7, CHO4655: ATAGCGAGCGTTGTACCGGC  P8, CHO4656: ACCTCCACGCAGCTATTGCC  P9, CHO4657: TTGACACGCCATCGAAGGAA  P10, CHO4658: CCCAAATCTGGGGCGTTACT |
| *isp7Δ::NAT^R^* | P1, CHO4616: CACAGTCGGCTTACCGAAGT  P2, CHO4617: AACCATACACTCAGCGCACAGCCTGGGTGGATTGAAAGGA  P3, CHO4618: TCCTTTCAATCCACCCAGGCTGTGCGCTGAGTGTATGGTT  P4, CHO4619: CACGAGGCCGATGAACTCATTGTGCTGGAATTCGCCCTT  P5, CHO4620: AAGGGCGAATTCCAGCACAATGAGTTCATCGGCCTCGTG  P6, CHO4621: GGGAGGAGGAATGCGATCAG  P7, CHO4651: GGGGGTACTAAGGGATGGGT  P8, CHO4652: GACGTGTGAACGCTGAGGTA  P9, CHO4653: CACTGCCGTTTCAACAGTGG  P10, CHO4654: ATGTTGGCCCGGAATCATGT |
| Common screening primers | CHO455: GGGTCTGTCAAAGGTCGAGCAAC  CHO456: GTGTTGACCCAGAATGGTTAGC  CHO4646: GTCGAGTGACGTTGCTGCTA  CHO4647: TGTGCGCTGAGTGTATGGTT  CHO4476: GGCGAATGGGCTGACCGCTT  CHO4396: CCAAGCGGCCGGAGAACCTG |
| qRT-PCR | CNE01720 P1, CHO5288: GCCCAGAAATGGCTTGAGGA  CNE01720 P2, CHO5289: GAGCCCTCCAGAGAGTACCA  CNE01730 P1, CHO5290: GATGATGCGCAGCGAAATGT  CNE01730 P2, CHO5291: CACGCGACCAACCAAAGATG  CNE01740 P1, CHO5292: GATCACGGAGACACTCTGGC  CNE01740 P2, CHO5293: CATTGAGTGGCCTTCGTCCT  GPD1 P1, CHO5294: TTGTTGCTGTCAACGACCCT  GPD1 P2, CHO5295: TTGAAGCGACCATGTGTGGA |

**Note:**

a. The 5'- region was amplified with primers P1 and P2, the 3'-region was amplified with P5 and P6, and the cassette of selection marker was amplified with P3 and P4. PCR fusion using P1 and P6 was used to create the final full-length deletion cassette.

b. P7 was used with one common screening primer (CHO455 for URA5, CHO4646 for *NAT^R^*, CHO4476 for *NEO^R^*) to screen for 5' correct insertion of the knockout construct, while P8 used with one common screening primer (CHO456 for URA5, CHO4647 for *NAT^R^*, CHO4396 for *NEO^R^*) for 3' correct insertion. P9 and P10 were used to screen for the absence of target ORF.
